# Urinary SPP1 has potential as a non‐invasive diagnostic marker for focal segmental glomerulosclerosis

**DOI:** 10.1002/2211-5463.13704

**Published:** 2023-09-27

**Authors:** Qinglin Ye, Guiling Xu, Chao Xue, Shuting Pang, Boji Xie, Guanwen Huang, Haoyu Li, Xuesong Chen, Rirong Yang, Wei Li

**Affiliations:** ^1^ Department of Nephrology The Second Affiliated Hospital of Guangxi Medical University Nanning China; ^2^ Centre for Genomic and Personalized Medicine Department of Immunology School of Basic Medical Sciences Guangxi Medical University Nanning 530021 China

**Keywords:** biomarker, diagnosis, focal segmental glomerulosclerosis, non‐invasive, SPP1, urine

## Abstract

Focal segmental glomerulosclerosis (FSGS) is a type of chronic glomerular nephropathy showing characteristic glomerular sclerosis, diagnosed by kidney biopsy. However, it is difficult and expensive to monitor disease progression with repeated renal biopsy in clinical practice, and thus here we explored the feasibility of urine biomarkers as non‐invasive diagnostic tools. We downloaded scRNA‐seq datasets of 20 urine cell samples and 3 kidney tissues and obtained two gene lists encoding extracellular proteins for bioinformatic analysis; in addition, we identified key EP‐Genes by immunohistochemical staining and performed bulk RNA sequencing with 12 urine samples. We report that urine cells and kidney cells were correlated. A total of 64 EP‐Genes were acquired by intersecting genes of distal tubular cluster with extracellular proteins. Function enrichment analysis showed that EP‐Genes might be involved in the immune response and extracellular components. Six key EP‐Genes were identified and correlated with renal function. IMC showed that key EP‐Genes were located mainly in tubules. Cross verification and examination of a urine RNAseq dataset showed that SPP1 had diagnostic potential for FSGS. The presence of urine SPP1 was primarily associated with macrophage infiltration in kidney, and the pathogenesis of FSGS may be related to innate immunity. Urinary cells seemed to be strongly similar to kidney cells. In summary, SPP1 levels reflect renal function and may have potential as a biomarker for non‐invasive diagnosis of FSGS.

## Graphical abstract

We explored the feasibility of urine biomarkers as a non‐invasive diagnostic tool for focal segmental glomerulosclerosis (FSGS), based on data from single cell sequencing datasets and gene lists encoding extracellular proteins. In addition, we performed bulk RNA sequencing of 12 urine samples. Our results indicate that urinary secreted phosphoprotein 1 of FSGS can reflect renal function and may serve as a marker for non‐invasive diagnosis.

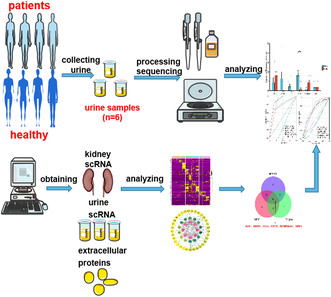

**Abbreviations**
ALB: albumin
APOE: apolipoprotein E
AUC: area under the curve
CLU: clusterin
CST3: cystatin C
DEGs: differentially expressed genes
EP‐Genes: extracellular protein genes
FSGS: focal segmental glomerulosclerosis
GEO: Gene Expression Omnibus
GFR: glomerular filtration rate
GO: Gene Ontology
IHC: immunohistochemistry
KEGG: Kyoto Encyclopedia of Genes and Genomes
MCC: Maximal Clique Centrality
MCODE: Molecular Complex Detection
PPI: protein–protein interaction
ROC: receiver operating characteristic
scRNA‐seq: single cell RNA sequencing
SERPINA1: serpin peptidase inhibitor
SPP1: secreted phosphoprotein 1

Focal segmental glomerulosclerosis (FSGS) is a type of chronic glomerular nephropathy showing characteristic glomerular sclerosis and its gold standard is a kidney biopsy [1, 2]. Relative to other glomerular disease diagnoses, the prevalence of FSGS appears to be increasing worldwide and it is a major cause of end‐stage kidney disease [3]. Because the pathological features of FSGS are special, patients sometimes need repeated biopsies for an accurate diagnosis [4]. Therefore, it is difficult to monitor the disease progression with repeated renal biopsy in clinical practice. At the same time, kidney biopsies are relatively expensive. A specialized physician is required to perform the procedure and a list of contraindications (bleeding diathesis, presence of isolated natural kidneys, uncontrolled severe hypertension, anticoagulants or antiplatelet agents) is needed that limit the availability of percutaneous kidney biopsy as a diagnostic tool [5]. Besides, there are no new biomarkers that are superior to the estimated glomerular filtration rate (GFR) or albuminuria [6]. However, GFR or albuminuria will only elevate after patients have developed renal insufficiency and cannot be used to predict and/or monitor disease progression [7, 8, 9]. Therefore, it is imperative to explore reliable biomarkers that reflect disease activity and can be used to monitor treatment response and progression, as well as determine disease prognosis.

Urine is an easily accessible biological matrix that can be used to identify novel biomarkers [10]. As a specimen, urine has several advantages over serum or plasma. Urine can be collected easily and non‐invasively and does not contain other components that may interfere with blood analysis [11]. Recent research suggests that the urinary matrix metallopeptidase‐9/neutrophil gelatinase‐associated lipocalin ratio can be used as a biomarker for FSGS in children with nephrotic syndrome [12] and urinary neutrophil gelatinase‐associated lipocalin can be used as a marker of progression of renal impairment in children with FSGS [13]. In addition, brain‐derived neurotrophic factor mRNA in urine cells could serve as a potential chronic kidney disease biomarker [14] and urinary mRNA has been considered to be as non‐invasive and potential biomarkers of acute rejection (AR) [15, 16, 17, 18] and diabetic kidney disease [19]. Consequently, it is of great interest to look for potential urinary biomarkers. Some studies had been shown that extracellular proteins can be used as markers of kidney disease diagnosis and progression [20, 21]. Extracellular proteins can become bright biomarkers by secreting into body fluid. No studies have been conducted that combine extracellular proteins with urine. In recent years, with the development of molecular biology technology, single cell sequencing is beneficial for analyzing the cellular heterogeneity of complex tissues [22]. On the other hand, abnormalities in any of these cell types might lead to kidney disease, prompting research to reduce gene expression levels to specific kidney cell types [23]. Various different cell types interfere with each other and affect kidney diseases progression. Currently, most cases of nephrotic FSGS are treated with immunosuppressive agents, but the underlying immune dysregulation and involved immune cells are not fully understood. Furthermore, the pathogenetic role of FSGS is unclear. It is not known whether this effect is exerted through direct cellular interactions between immune cells and epithelial cells or through secreted factors at a more distant distance [24]. Thus, compared with traditional methods, it is more precise and helpful for us to explore biomarkers from the view of cell type‐specificity at the single cell level.

In sum, we hypothesized that the extracellular proteins enter the urine as a result of kidney injury and this is the first time urine has been associated with extracellular proteins to explore urine biomarkers. We combined urinary single cell sequencing with kidney single cell sequencing to trace the origin of urine cells. We also explored the level of key extracellular protein genes (EP‐Genes) expression and the relationship with time in urine aiming to determine ‘urinary‐kidney specific biomarkers’ that better identify the significant cell population and reflect the *in situ* molecular changes in FSGS.

## Materials and methods

### Acquisition of data and processing

The single‐cell datasets GSE176465 and GSE131685 were downloaded from the Gene Expression Omnibus (GEO) database (https://www.ncbi.nlm.nih.gov/geo), which contained three healthy human kidneys and 20 urine cell samples from FSGS. The gene expression profile datasets GSE125779 included eight kidney tissues of FSGS and eight healthy kidney tissues of control, and GSE121211 included five kidney tissues of FSGS and five healthy kidney tissues of control were also obtained. Two expression profile datasets were analyzed by using the GEO2R online analysis tool provided with the GEO database. Next, we integrated and visualized GSE176465 and GSE131685, respectively, in the same manner as the method shown below. We used the r package harmony [26] to integrate single cell RNA sequencing (scRNA‐seq) data. The scRNA‐seq data were normalized by log‐normalization and the FindVariableFeatures function was used to find highly variable genes. To identify cell type markers, we used Seurat's ‘FindAllMarkers’ function.

### Integration of kidney single cell data with urine scRNA‐seq

**Fig. 1:**
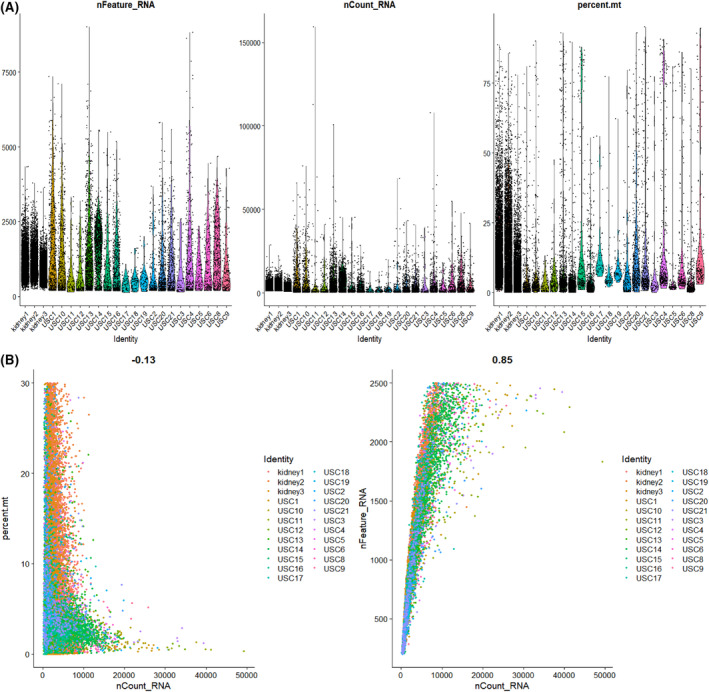
Quality control (QC) of human kidney and FSGS urine single cell datasets from GSE131685 and GSE176465. (A) Scatterplot illustrating the number of genes, unique molecular identifers (UMIs) and the percentage of mitochondrial genes in each cell of three kidney samples and 20 urine cell samples. (B) The relationship between the percentage of mitochondrial genes and the mRNA reads, together with the relationship between the amount of mRNA and the reads of mRNA.

We used the MergeSeurat [25] function to merged the three kidney datasets and 20 urine cell datasets. According to the median number of genes and the percentage of mitochondrial genes (Fig. 1A), cells with < 200 and > 2500 genes and cells with 30% mitochondrial RNA reads were excluded. After quality control, the relationship between the percentage of mitochondrial genes and the mRNA reads was detected and visualized, together with the relationship between the number of mRNAs and the reads of mRNA (Fig. 1B). We used the r package harmony [26] to integrate scRNA‐seq data and the data were normalized by log‐normalization. Then, the cells were clustered using the FindNeighbors and FindClusters functions (set resolution = 0.3) to obtain each type of cell and classified into 12 different cell types. Finally, we used the ‘FindAllMarkers’ function to identify cell type markers.

### Screening of EP‐Genes

Cell clusters are formed according to gene expression similarity [27]. We found that, in cluster 10, urine cells derived from urine cell sequencing data co‐aggregated with kidney cells derived from renal cell sequencing. Therefore, we selected genes in cluster 10 (distal tubular cells). Next, we chose 1006 ScRNA‐differentially expressed genes (DEGs) |log fold change (logFC)| > 0.25, adjusted *P* < 0.05) [28] were differentially expressed in at least one type of cell. Tow lists of extracellular protein genes were required from the Human Protein Atlas (HPA) database [29] and the UniProt database (GO:0005576) [30]. We intersected two extracellular protein genes with ScRNA‐DEG‐genes by drawing a Venn diagram and acquired EP‐Genes for in depth analyses.

### Pathways and disease enrichment analysis of EP‐Genes

We conducted Gene Ontology (GO) (http://geneontology.org) and Kyoto Encyclopedia of Genes and Genomes (KEGG) (https://www.genome.jp/kegg) pathway enrichment analysis of the 64 EP‐Genes using the Database for Annotation, Visualization and Integrated Discovery (DAVID; https://david.ncifcrf.gov/home.jsp). At disease level, we acquired disease enrichment information from the Search Tool for the Retrieval of Interacting Genes online database (STRING; https://string‐db.org).

### Protein interaction network analysis and recognition of the key extracellular protein genes

STRING was applied to construct protein–protein interaction (PPI) of 64 EP‐Genes, which was visualized by using cytoscape, (version 3.9.1) [31]. Next, we obtained the top 10 node genes respectively by the maximal clique centrality (MCC) method and “Degree” methods. Meantimes, we explored important genes in the PPI network using Molecular Complex Detection (MCODE) in Cytoscape. Eventually, we considered the overlap of genes obtained by the three methods as the key extracellular protein genes.

### Gene expression and clinical correlation analysis

The Nephroseq V5 tool (Nephroseq V5; https://www.nephroseq.org/resource/login.html) was used to identify the expression level of key EP‐Genes [albumin (ALB), apolipoprotein E (APOE), clusterin (CLU), cystatin C (CST3), serpin peptidase inhibitor (SERPINA1) and secreted phosphoprotein 1 (SPP1)] in kidney tissue of FSGS. In addition, we downloaded clinical information in the patients with FSGS and used “ggplot2” package [32] to visualize.

### Validation of key EP‐Genes and diagnostic value analysis by using public datasets

To verify the reliability of key EP‐Genes, we downloaded the gene expression profile GSE125779 and GSE121211 from the GEO database and verified the mRNA expression level of key EP‐Genes. To evaluate the diagnostic value of the biomarkers, we plotted the receiver operating characteristic (ROC) curve and calculated the area under curve (AUC) using the “proc” package [33]. *P* < 0.05 was considered statistically significant.

### Immunohistochemistry (IHC) validation

Renal tissue sections including six patients of FSGS and three kidney para‐cancerous samples were obtained from the Second Affiliated Hospital of Guangxi Medical University. Ethical approval for this study was obtained from The Human Research Ethics Committee of the Second Affiliated Hospital of Guangxi Medical University. All patients provided written and verbal informed consent. Then tissue sections were incubated overnight at 4 °C with rabbit monoclonal antibodies against ALB (16475‐1‐AP; dilution 1 : 100; ProteinTech, Rosemont, IL, USA), APOE (AF5178; dilution 1 : 150; Affinity, Cincinnati, OH, USA), CLU (DF6421; dilution 1 : 100; Affinity), CST3 (DF6457; dilution 1 : 120; Affinity), SERPINA1 (DF6182; dilution 1 : 100; Affinity) and SPP1 (BS‐0026R; dilution 1 : 150; BIOSS, Woburn, MA, USA). This was followed by incubation with secondary antibodies at room temperature for 20 min. Tissue sections were dyed with hematoxylin and then counterstained, dehydrated and mounted. IHC results were analyzed using imagej (NIH, Bethesda, MD, USA).

### Urine samples processing and urinary cell RNA‐Seq

We enrolled three patients who were diagnosed FSGS by renal biopsy and three healthy individuals as control samples. Two urine samples were collected from each individual. Exclusion criteria were malignancies, severely impaired hepatic function or other rheumatic diseases. All biopsies were assessed by pathologists who were blinded to patient outcomes. None of the patients received the biopsy procedure and therapy before the collection of urine samples.

Once the samples were obtained, they were immediately transported on ice to the laboratory for processing. All steps were performed on ice or at 4 °C. Once the urine volume was measured, the sample was transferred into a 50‐mL conical tube and centrifuged at 490 ***g*** for 10 min. The supernatant was carefully removed, leaving approximately 1 mL in the tube. Cells then were suspended in 1× Dulbecco's phosphate‐buffered saline and centrifuged at 2000 r.p.m (485 ***g***). for 5 min. This process was repeated twice. cDNA libraries were prepared in accordance with the manufacturer’ protocol (HTP OneStep RNAseq Kit for 96 wells, Singleron, China). In brief, cell lysis and RNA capture were performed by using 10 μL of cell lysis buffer. cDNA was amplified and purified using OneStep Buffer.

Next, we performed RNA‐Seq of six urine samples. This study was approved by the ethics commission of Guangxi Medical University Ethics Committee and was performed under the ethical principles of the Declaration of Helsinki [2022‐KY (0623)].

### Urinary cell sequencing data analysis and validation of key EP‐Genes in urine

We used the ‘limma’ package [34] for differential expression analysis of urine RNA‐seq data. Genes significantly DEGs in urine with log_2_FC > 1 and *P* < 0.05 were considered for supervised downstream analysis. Then we investigated expression of key EP‐Genes between the FSGS and healthy groups in urine. Finally, we used ROC analysis to evaluate their diagnostic value in urine.

## Results

### Kidney cells co‐cluster with urinary cells

**Fig. 2:**
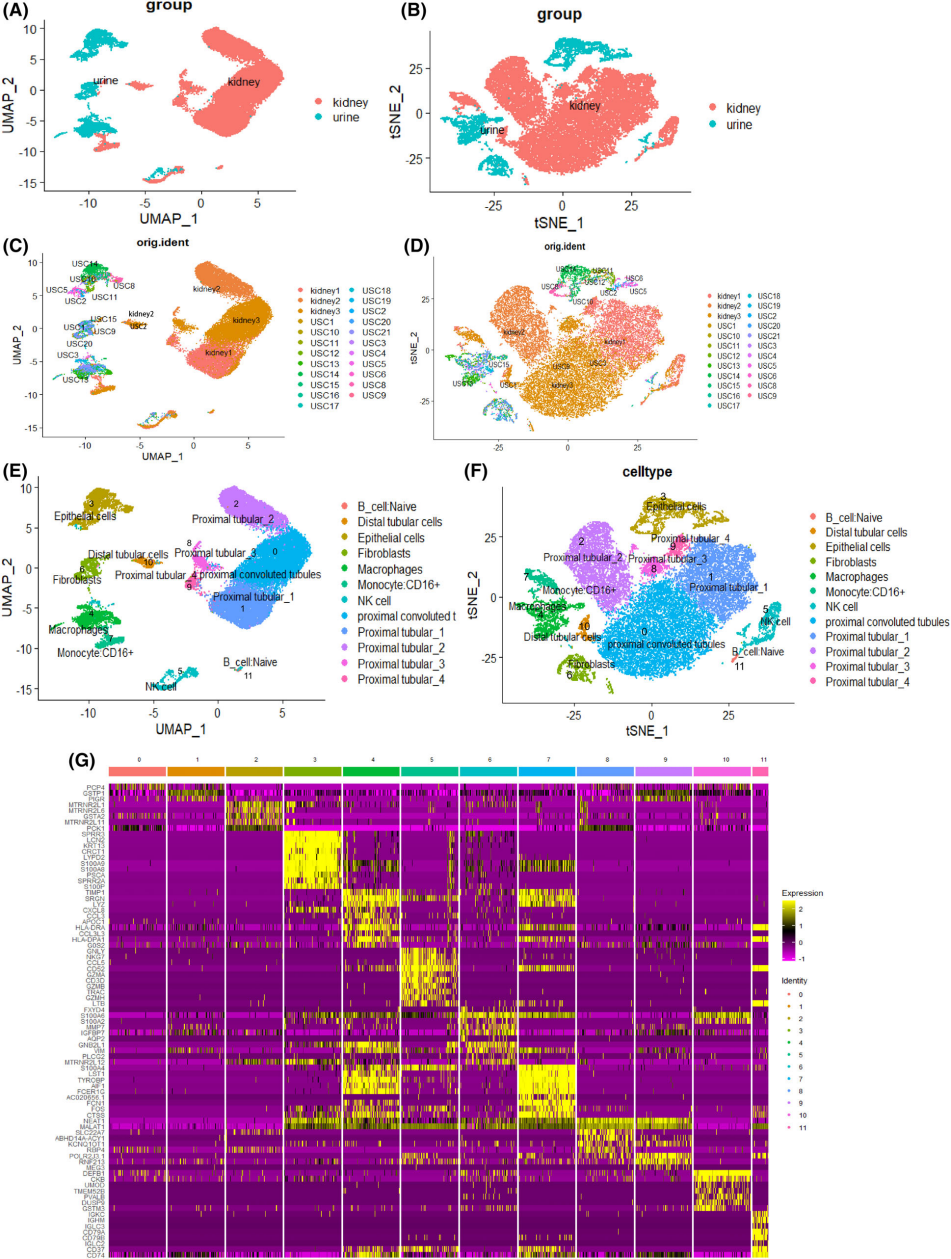
Integration of FSGS urine single cell with human kidney single cell datasets. (A, B) UMAP and tSNE of harmony‐based integration of urinary and kidney cells colored by the sample of origin. Blue indicates the urine origin and pink shows the cells originated from kidney. (C, D) The same t‐SNE and UMAP plots with a different color for each sample. (E, F) t‐SNE and UMAP plots of urine and kidney single‐cell aggregate data from 20 urine samples and three kidney samples revealing 12 individual cell clusters and cell types. (G) Principal component heatmap plot revealing 10 most highly expressed genes in each of 12 clusters (vertical columns), with each row representing one gene, with high expression (yellow), intermediate expression (purple) and low expression (black).

**Fig. 3:**
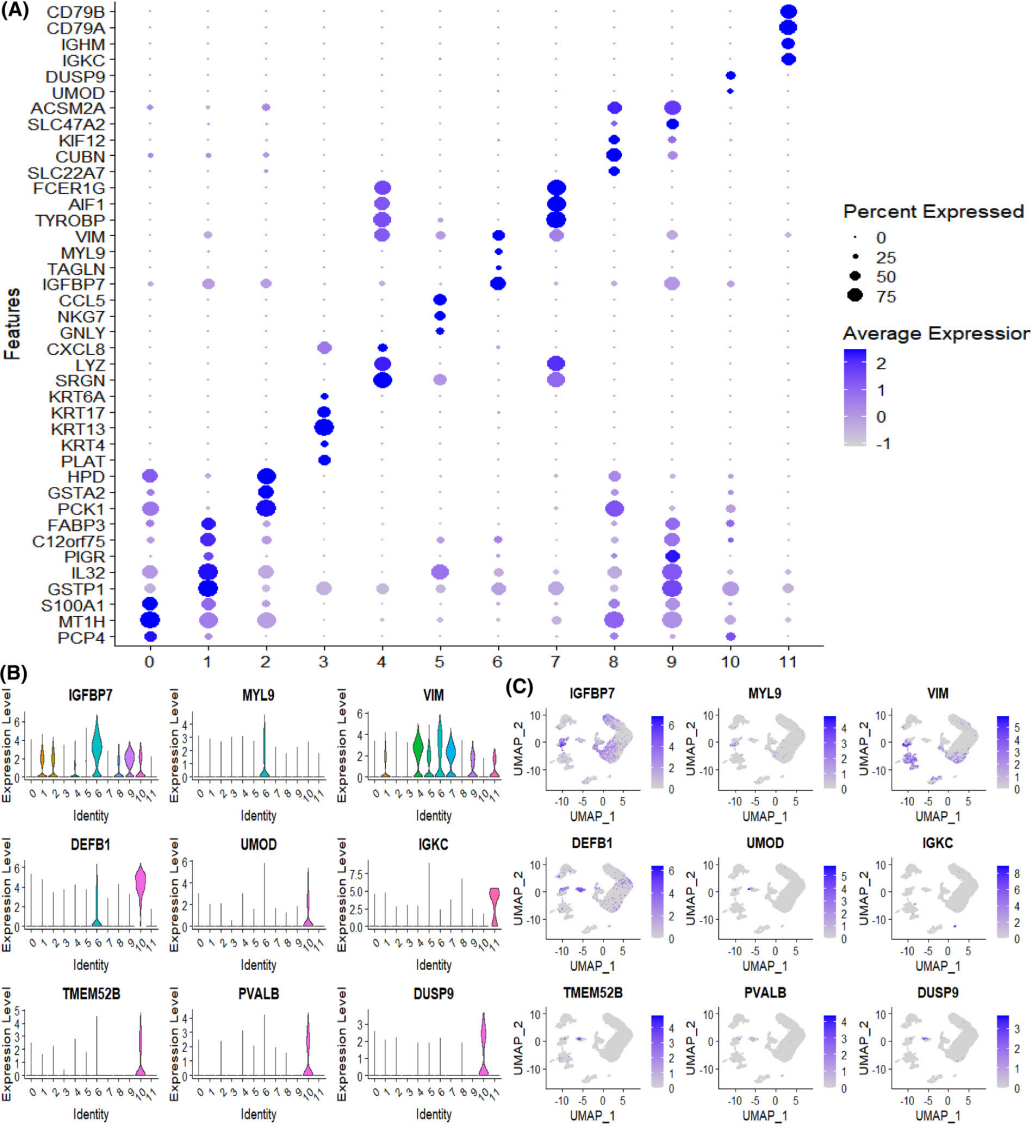
scRNA‐seq identified genes specifically expressed. (A) Bubble dot plots of the top cell type‐specific DEGs in the integrated clusters of urine and kidney samples. The size of the dot indicates the expression percentage and the darkness of the color indicates average expression. (B) UMAP plots show the expression of marker genes (cluster 10 and cluster 6). (C) The violin plots show the distribution and relative maker genes expression across cell types (cluster 10 and cluster 6).

To investigate the origin of urine cells and their relationship to kidney cells, we integrated our FSGS urine single‐cell dataset with human kidney single‐cell datasets, which included 20 urine cell samples and three kidney samples, to explore ‘urinary‐kidney specific genes’. We identified 12 clusters using harmony integration (Fig. 2A–F) and marker genes are listed in Fig. 3A (Figs S1–S3). We selected genes according to the classification of cell groups, and the expression of the top 10 marker genes was visualized using a cluster heatmap (Fig. 2G). We found that there was a striking similarity between urine cells and kidney cells. For example, urine cells and kidney cells of cluster 10, which was positive for the distal tubular cells markers DEFB1, UMOD, TMEM52B, PVALB, DUSP9 and PPP1R1A (Fig. 3B,C), had clustered closely. At the same time, we also found urinary cells had clustered closely with kidney cells in cluster 6 that had expressed the fibrosis marker genes markers IGFBP7, MYL9 and VIM (Fig. 3B,C). We inferred that it was possible for fibrosis cells and distal tubular cells to be shed into the urine with the progression of FSGS. In addition, we observed that urine cells co‐clustered with kidney cells, indicating that urine cells were related to kidney cells to some extent and also that urine can be used to assess and diagnose FSGS.

### Screening of EP‐Genes

**Fig. 4:**
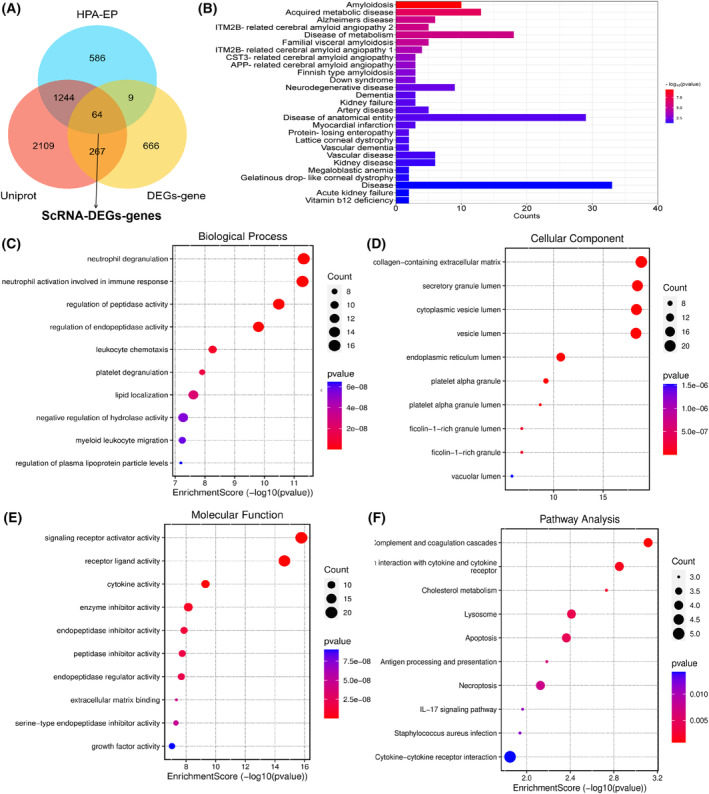
DEGs encoding extracellular proteins genes (EP‐Genes) and Function enrichment analysis of EP‐Genes. (A) Venn diagram of 64 EP‐Genes. obtained based on the intersection of HPA database‐extracellular proteins, ScRNA‐DEGs‐genes and UniProt. (B) Disease enrichment analysis of EP‐Genes. (C–E) GO enrichment analysis of EP‐Genes in BP, CC and MF processes (BP, biological process; CC, cellular component; MF, molecular function). (F) KEGG enrichment analysis of EP‐Genes. In the dot plot, the color represents the *P*‐value, and the size of the spots represents the gene number.

Sixty‐four EP‐Genes were obtained based on the intersection of 1903 genes from HPA database‐extracellular proteins, 1006 ScRNA‐DEGs‐genes and 3685 genes from UniProt using a Venn diagram (Fig. 4A, Data S1).

### Pathways and disease enrichment analysis of EP‐Genes

**Fig. 5:**
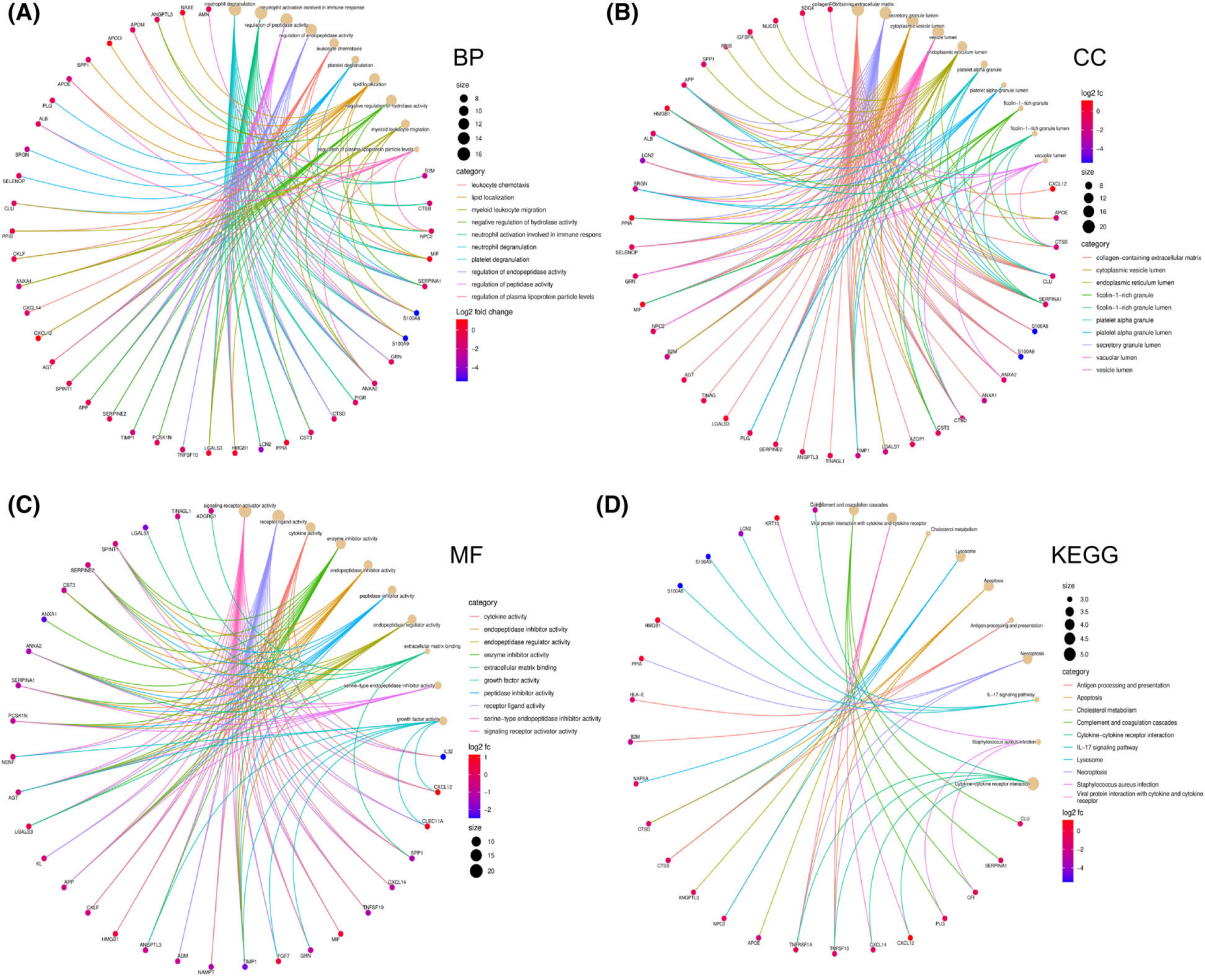
Function enrichment analysis of EP‐Genes by GO. (A–C) Circle graph showing the top 10 pathways of BP, CC and MF, respectively. (D) Circle graph showing the top 10 pathways of KEGG. Different colors represent different pathways, and the DEGs in the pathway are displayed.

GO, KEGG and diseases enrichment were used to investigate the mechanisms of FSGS. Disease enrichment pathway analysis indicated that 64 EP‐Genes associated with acute kidney failure, kidney failure and kidney diseases (Fig. 4B). The GO analysis results showed that the 64 EP‐Genes were mainly enriched in neutrophil degranulation, neutrophil activation involved in the immune response, regulation of peptidase activity and regulation of endopeptidase activity (Fig. 4C), whereas the cellular component category mainly participated in the collagen‐containing extracellular matrix, secretory granule lumen and cytoplasmic vesicle lumen (Fig. 4D). In terms of molecular function, EP‐Genes were mainly involved in signaling receptor activator activity and receptor ligand activity (Fig. 4E). KEGG enrichment pathway analysis showed that EP‐Genes were mainly enriched in complement and coagulation cascades, cytokine–cytokine receptor interaction, and antigen processing and presentation (Fig. 4F). Moreover, the top 10 pathways of BP, CC, MF and KEGG are shown in Fig. 5.

### PPI network analysis and key EP‐Genes recognition

**Fig. 6:**
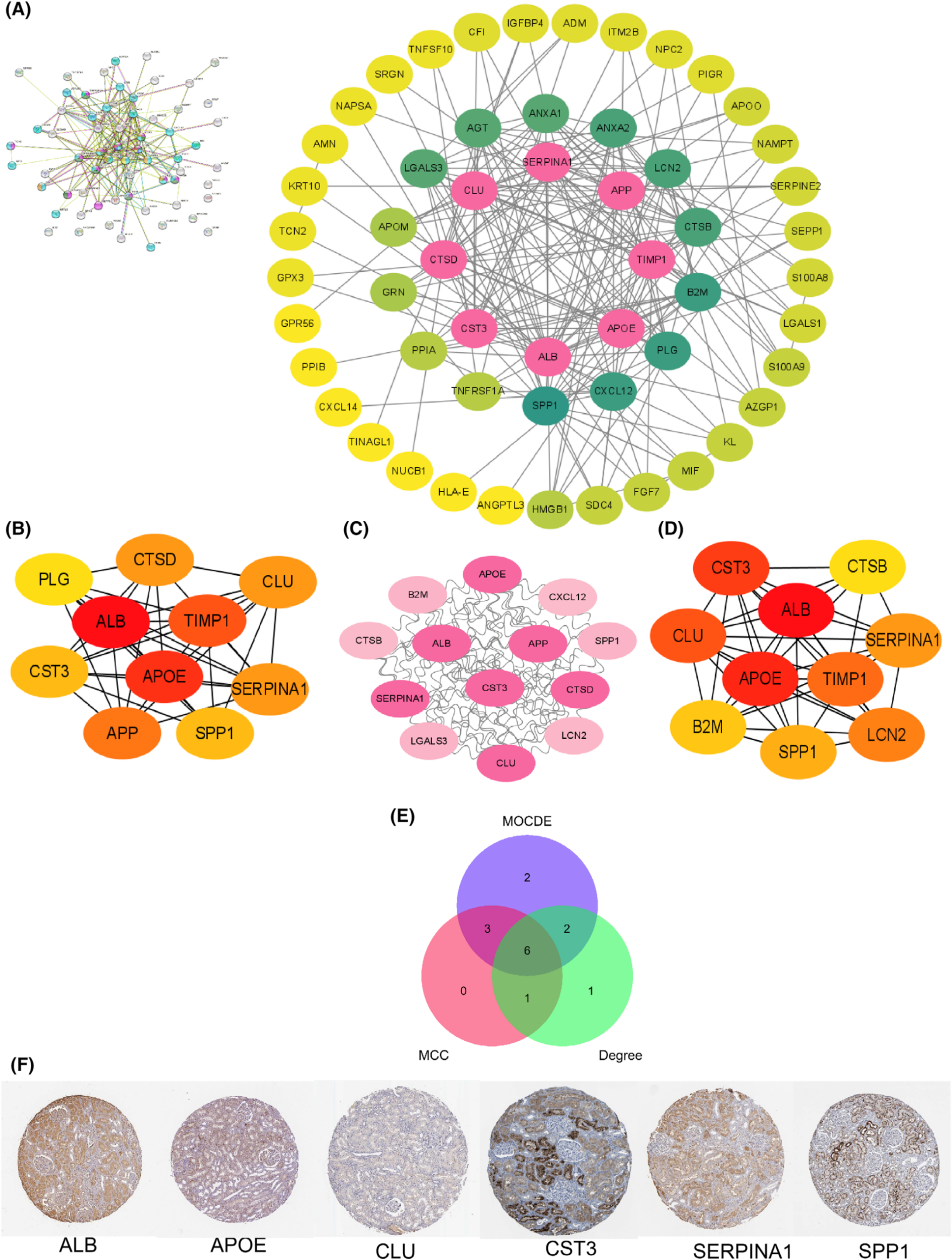
Identification of key EP‐Genes. (A) Construction of the PPI network of 64 EP‐Genes by STRING database. (B) Top 10 genes were obtained by calculating the scores according to the MCC method. (C) In total, 13 candidate genes were identified from EP‐Genes basing on the score sorted by the MCODE method using cytoscape. (D) Top 10 genes were selected according to the score calculated by the Degree method. (E) Venn diagram showing six key EP‐Genes (ALB, APOE, CLU, CST3, SERPINA1 and SPP1) obtained by the intersection of the methods (MCC, Degree and MCODE). (F) IHC staining of six EP‐Genes; from the Human Protein Atlas (https://www.protein Atlas.org.

Based on STRING and cytoscape, the PPI network of 64 EP‐Genes was constructed and visualized (Fig. 6A). Next, we used the MCC plugin of cytoscape to screened out 10 hub genes from EP‐Genes (Fig. 6B). Meanwhile, the top 10 candidate genes were obtained by calculating the scores according to the MCODE and Degree methods, respectively (Fig. 6C,D). Finally, the six key EP‐Genes (ALB, APOE, CLU, CST3, SERPINA1 and SPP1) were obtained through the intersection of the three methods (Fig. 6E). To verify the tissue‐specific gene, with the exception of CLU, IHC staining of the other five genes was performed, which is mainly activated in kidney (Fig. 6F).

### Correlation analysis of the six key EP‐Genes with the clinical features in FSGS

**Fig. 7:**
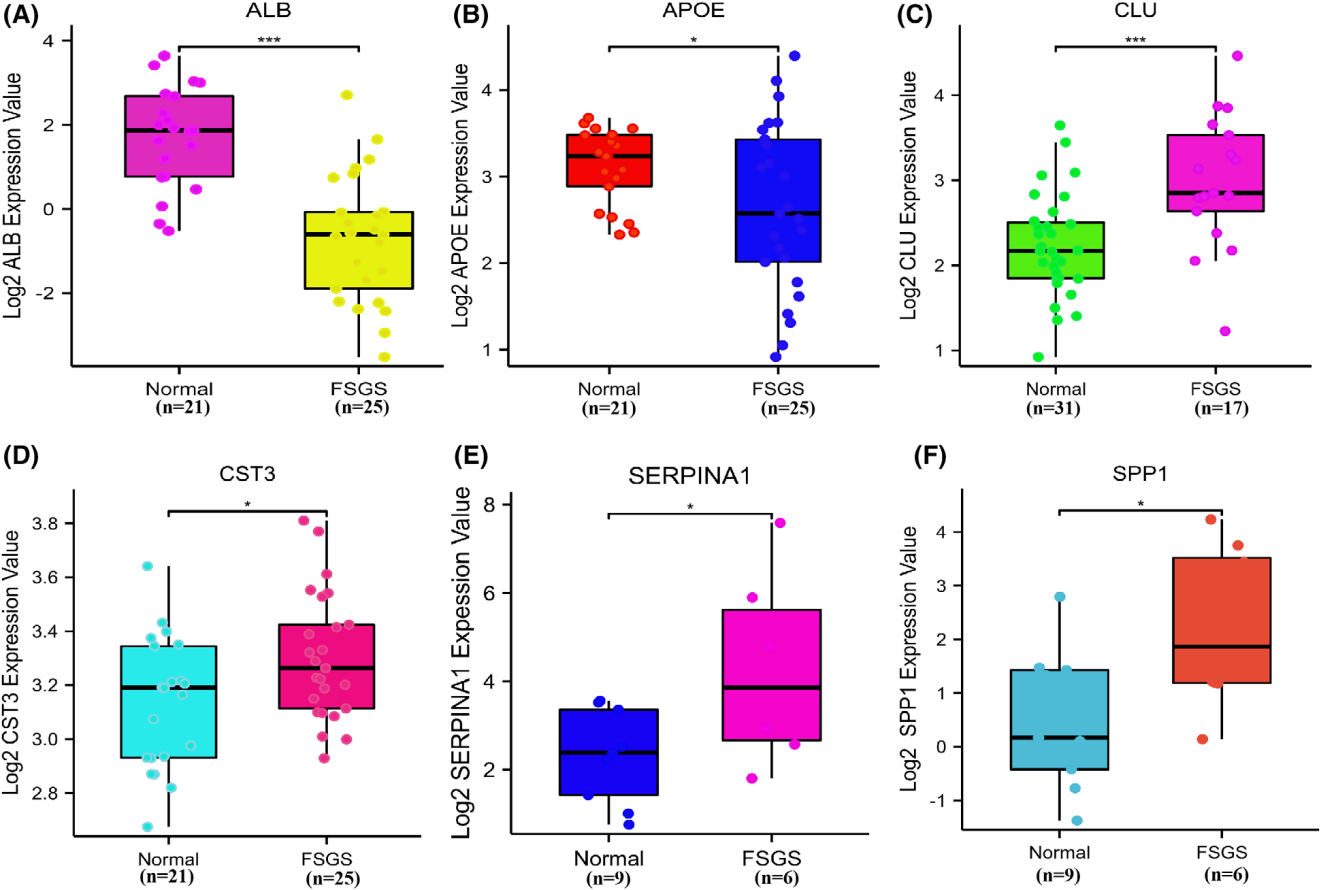
The expression of key EP‐Genesand data from nephroseq V5. (A–F) The expression level of ALB, APOE, CLU, CST3, SERPINA1 and SPP1 between FSGS and control groups using a *t*‐test (*P < 0.05 and ***P < 0.001) (*n* values, numbers of biologically‐independent replicates).

Six key EP‐Genes (ALB, APOE, CLU, CST3, SERPINA1 and SPP1) were identified as urine biomarkers. Based on the Nephroseq database, we explored the kidney tissue expression level of key EP‐Genes between the FSGS and normal groups (Fig. 7). Compared to the controls, there were significant differences in the expression levels of six genes in FSGS. ALB and APOE were upregulated in the normal group, whereas CLU, CST3, SERINA1 and SPP1 were significantly upregulated in the FSGS group.

**Fig. 8:**
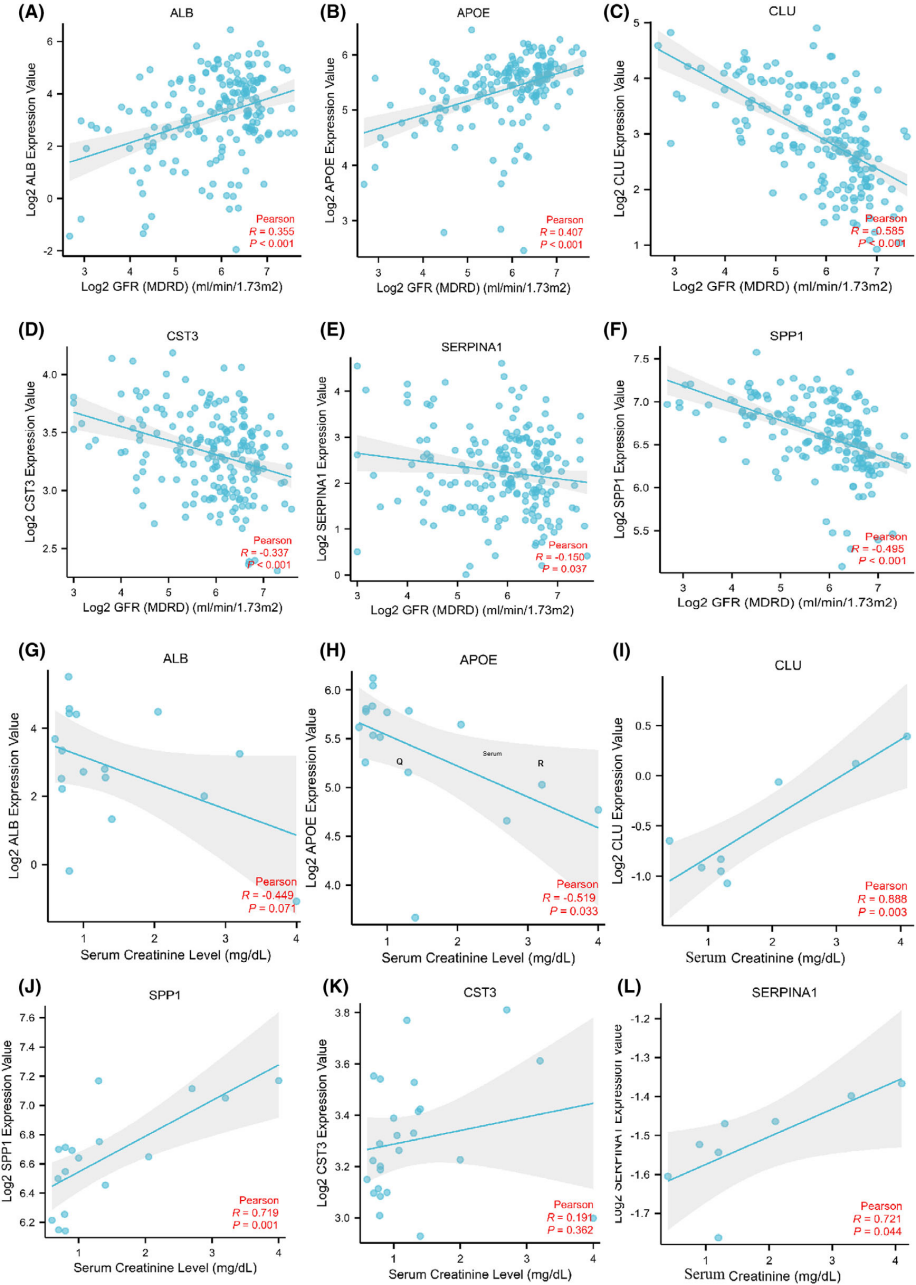
Correlation analysis of ALB, APOE, CLU, CST3, SERPINA1 and SPP1 expression with the clinicopathological features, including (A–F) glomerular filtration rate (GFR) and (G–L) serum creatinine level.

To further explore the clinical significance of the key EP‐Genes in FSGS, we performed correlation analysis of key EP‐Genes expression with several clinicopathological features based on the Nephroseq database. The results revealed that there was a significantly negative association between CLU/CST3/SERPINA1/SPP1 expression and GFR (Fig. 8A–F) and there was a significantly positive correlation between CST3/SPP1/SERPRINA1/CLU expression and serum creatinine level (Fig. 8G–L). These results indicated that high expression of ALB/SPP1/SERPRINA1/CLU might predict worse progression of FSGS. In addition, with the progression of FSGS, kidney cells may be shed into the urine and it was also possible for them to be highly expressed in urine.

### Validation of key EP‐Genes and diagnostic value analysis in validation groups

**Fig. 9:**
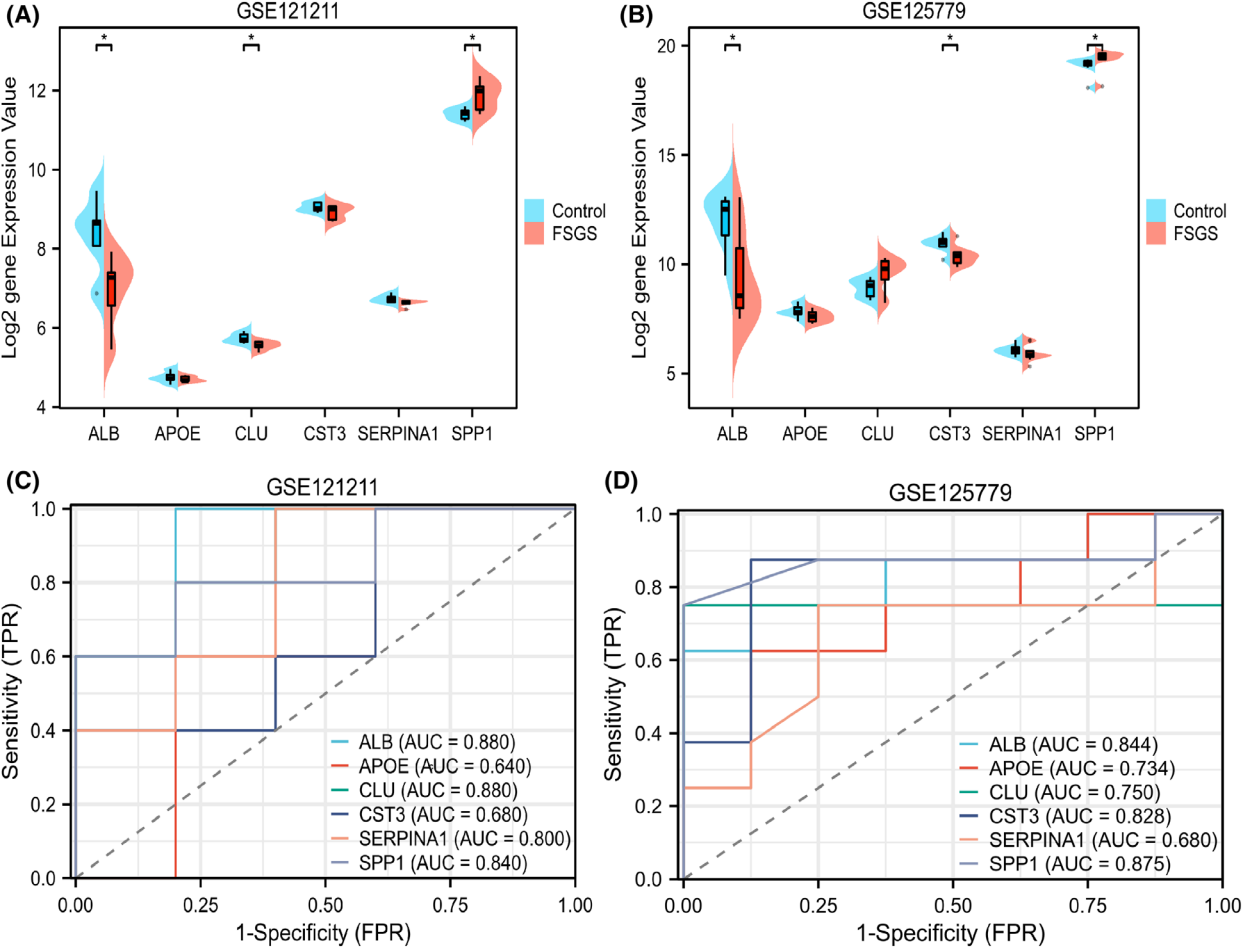
Key genes expression and diagnostic value analysis of the potential urine biomarkers in the kidney. The expression level of six key genes between FSGS and normal controls in (A) GSE121211 and (B) GSE125779. The diagnostic value of six key genes according to AUC value in ROC curve based on (C) GSE121211 (AUC = 0.880, 0.640, 0.880, 0.680, 0.800 and 0.840, respectively) and (D) GSE125779 (AUC = 0.844, 0.734, 0.750, 0.828, 0.680 and 0.875, respectively) (*P < 0.05).

The expression data from GSE125779 and GSE121211 were normalized. We found that the expression of SPP1 and ALB effectively distinguished FSGS from the normal group, whereas there were no significant differences for the other four genes. ALB was downregulated in the FSGS group and SPP1 was significantly upregulated in the FSGS group (Fig. 9A,B). The results were consistent with previous results.

The ROC curve is a useful tool for assessing the performance of a diagnostic test within the range of possible values for the predictor variable [35]. The results suggested that ALB, APOE, CLU, CST3, SERPINA1 and SPP1 had a high diagnostic value according to GSE121211 (AUC = 0.880, 0.640, 0.880, 0.680, 0.800 and 0.840, respectively) (Fig. 9C) and GSE125779 (AUC = 0.844, 0.734, 0.750, 0.828, 0.680 and 0.875, respectively) (Fig. 9D). It was worth noting that the AUC values of SPP1 and ALB were > 0.8 in both data sets.

### Localization and verification of key EP‐Genes in kidney

**Fig. 10:**
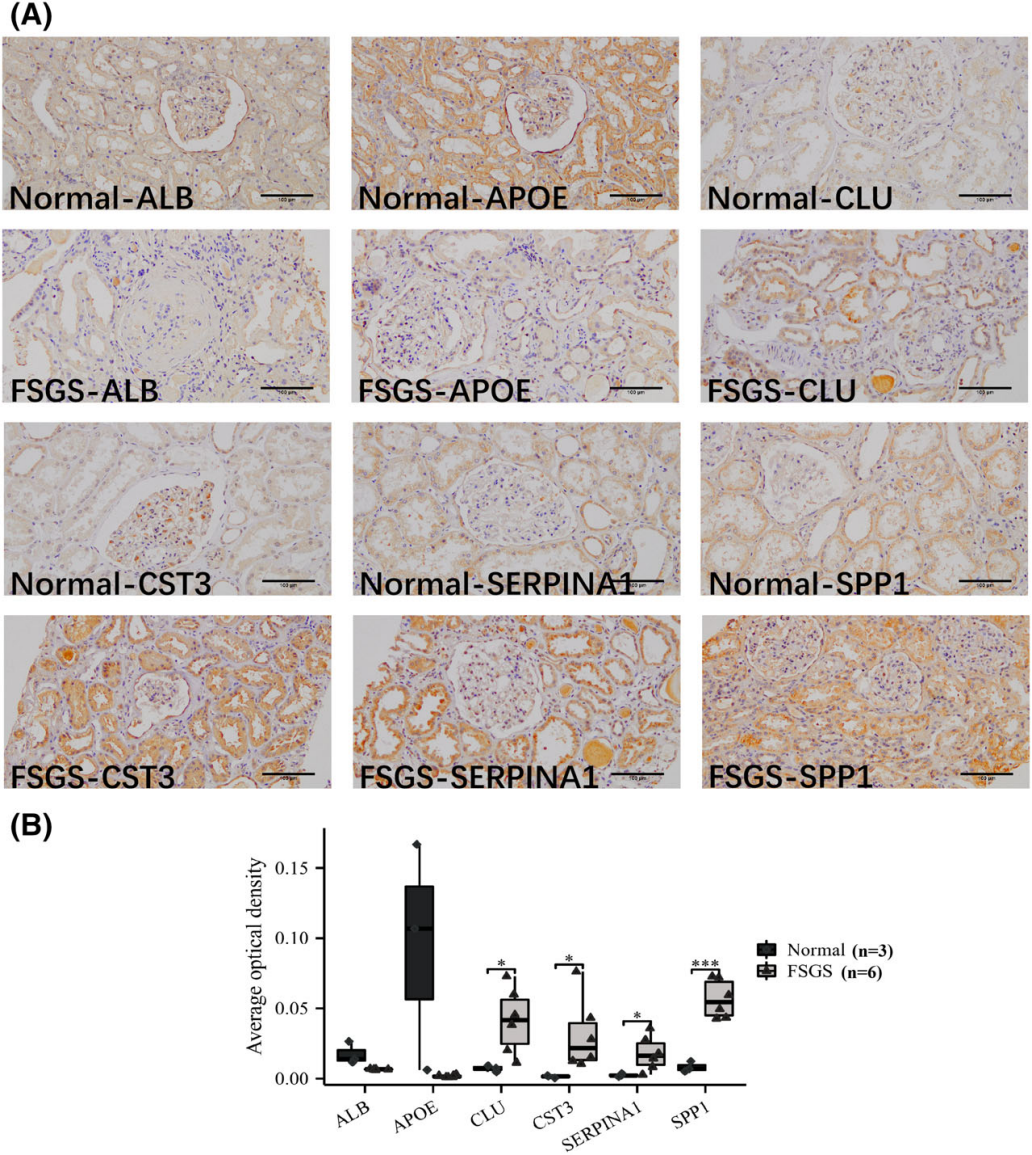
Validation and location analysis of six key EP‐Genes. (A) Immunohistochemical stain for six key EP‐Genes of the FSGS patients and controls. Original magnification, ×200. Scale bars = 100 μm. (B) Semi‐quantitative statistical comparison of Masson stained blue collagen area using a *t*‐test (*P < 0.05 and ***P < 0.001) (*n* values, numbers of biologically‐independent replicates).

The IHC results of kidney tissue showed that, compared to controls CST3/SERPINA1/CLU/SPP1 were highly expressed in patients of the FSGS group. ALB/APOE were not statistically significant in the FSGS and normal groups. Furthermore, we found that six key EP‐Genes were located mainly in tubular but not in glomerular compartments (Fig. 10).

### Urinary cell sequencing data analysis and validation of key EP‐Genes in urine

**Fig. 11:**
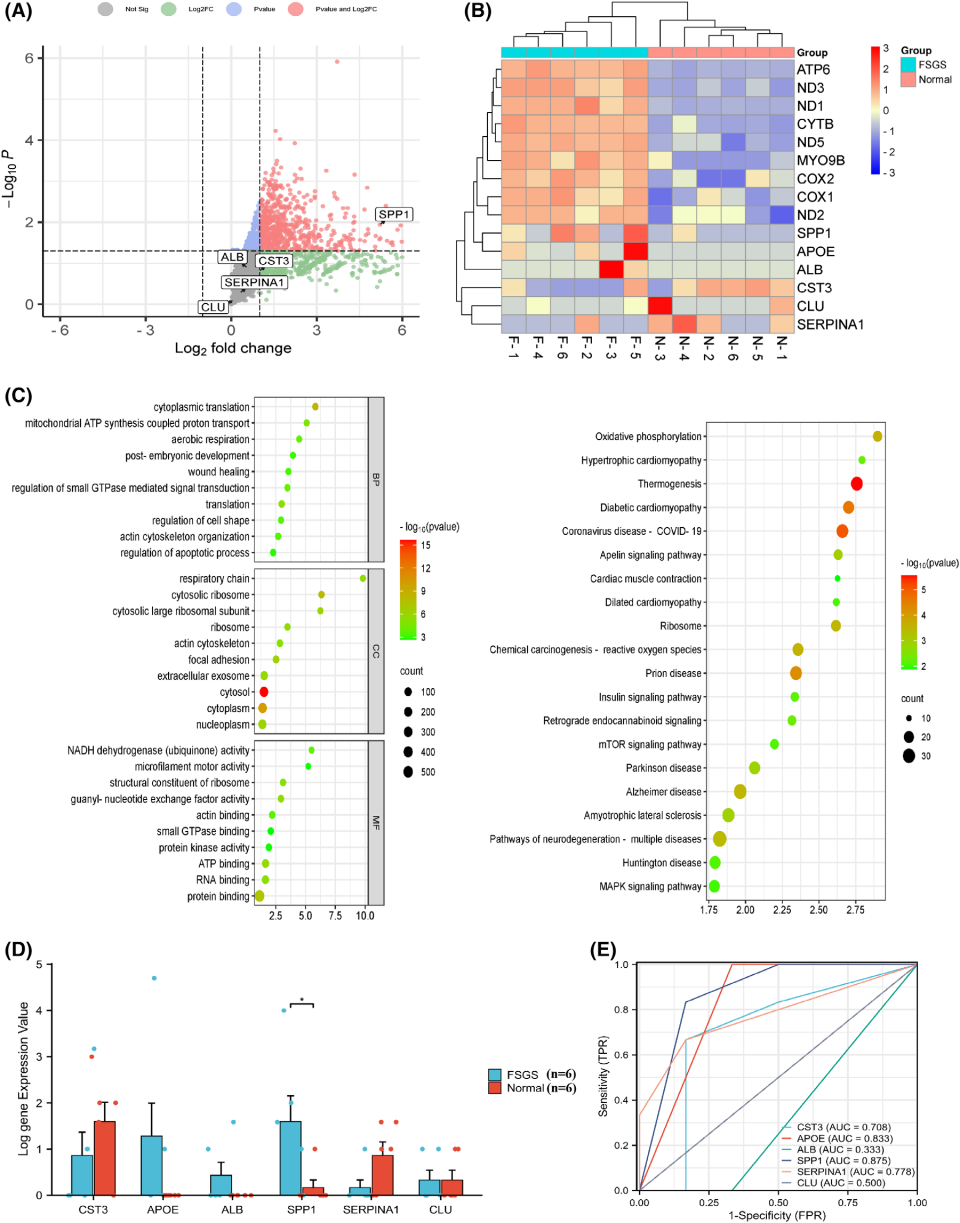
Urinary cell sequencing data analysis and Key EP‐Genes expression and diagnostic value analysis of FSGS in urine. (A) Volcano plot of DEGs between FSGS patients and control. Six key EP‐Genes were marked on the plot. (B) Expression heatmap of the top 14 DEGs and six key EP‐Genes. (C) GO and KEGG enrichment analyses of DEGs. (D) The expression level of six key EP‐Genes between FSGS and normal controls in urine using a *t*‐test. The error bars represent the SEM. (E) The diagnostic value of six key EP‐Genes in in the urine of patients (*P < 0.05) (*n* values, numbers of urine samples).

Eight hundred and twenty‐two DEGs were identified from the urine RNA‐seq dataset (Data S2) and the distribution of DEGs was depicted by the volcano plot shown in Fig. 11A. Subsequently, the top 14 DEGs and six key EP‐Genes were displayed using a heatmap (Fig. 11B). Then, we further performed KEGG and GO enrichment analyses, which indicated that urine DEGs primarily participated in cytoplasmic translation, cytosol, small substance binding and oxidative phosphorylation (Fig. 11C). Additionally, we compared the expression of key EP‐Genes in the control and FSGS urine samples. We only observed that the expression of SPP1 was significantly upregulated in FSGS group (Fig. 11D). Therefore, we hypothesized that the increase of SPP1 was proportional to kidney damage in the kidney and urine. In addition, SPP1 had the highest AUC value among six key EP‐Genes of FSGS (AUC = 0.875), which indicated SPP1 had SPP1 had a high diagnostic value (Fig. 11E). Furthermore, new research has shown that SPP1 expression was a marker of profibrotic macrophage populations [36] and it is possible for the presence of SPP1 in FSGS urine to be primarily related to infiltration macrophages into the kidney.

### SPP1 in urine is derived from renal cells

**Fig. 12:**
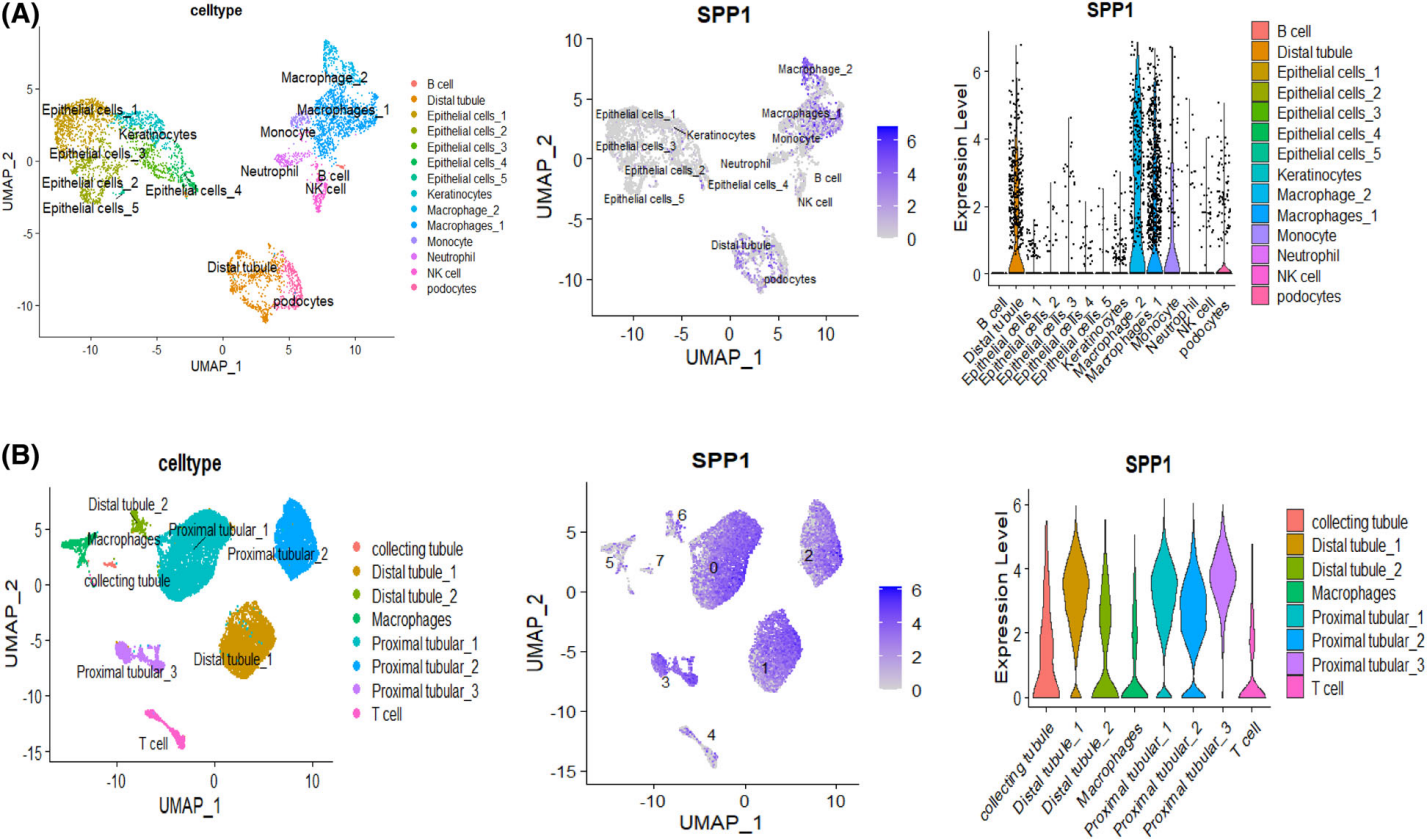
scRNA‐seq analysis validation of SPP1 in the GSE131685 and GSE176465 datasets, respectively. (A) The distribution of SPP1 in different cells and the relative expression level of SPP1 in identified cell types as a result of analyzing the human kidney scRNA‐seq from GSE131685. (B) The distribution of SPP1 in different cells and the relative expression level of SPP1 in identified cell types as a result of analyzing the FSGS urine scRNA‐seq from GSE176465.

To pinpoint the source of SPP1 in urine, we reanalyzed GSE176465 and GSE131685, respectively. The cell types of both datasets were identified by unsupervised clustering and expression analysis of marker genes following batch correction (Figs S2 and S3). We found that SPP1 expression was mainly significantly upregulated in the kidney cells, such as distal  tubular cell (Fig. 12A), which suggested that SPP1 was mainly secreted by renal tubular cell. Meantime, we further explored the cell type of SPP1 in urinary cells by analyzing GSE176465 dataset. The results indicated that SPP1 was specifically elevated in distal tubule cells, podocytes and macrophages of FSGS, which is consistent with the previous results (Fig. 12B).

## Discussion

scRNA‐seq enables the exploration of genetic features of urine and kidney at a single cell level and is a helpful tool for identifying novel biomarkers that indicate the development of diseases [37, 38]. A recent study showed that various types of renal cells could be detected in urine from the diabetic kidney disease patients [39], thus making it possible to develop non‐invasive method for pathological measurements. In the present study, we adopted scRNA‐seq data acquired from kidney and urine biopsies to explore ‘urinary‐kidney specific biomarkers’, connecting urine with extracellular proteins for the first time, and conducted research on the diagnosis and prognosis of FSGS.

Distal tubular cells have been shown to be closely related to kidney injury in various renal diseases, potentially indicating the development and progress of kidney injuries. For FSGS, distal tubular cells are critical for the development of tubulointerstitial lesions, which could be measured by the level of urinary biomarkers [40]. With the 64 DEGs identified from scRNA‐seq data, gene set enrichment analysis indicated that most of these genes were involved in immune responses and modulation of extracellular matrix, and were associated with kidney diseases. Further analysis showed that six key EP‐Genes (SPP1, APOE, CLU, CST3, SERPINA1 and ALB) could be critical for the above pathological/biological effect, considering that the expression level of these six genes significantly correlated with the progression of FSGS. Among these six genes, SPP1 showed great potential to serve as a marker indicating the progression of FSGS. Cells and genes detected from urine biopsies have been shown to be increased in patients suffering renal diseases, Urinary biomarkers that are increased by ischemia, oxidative stress, endotoxin, inflammation, and sepsis are correlated with the degree of interstitial tubular damage and reflect tubular damage [41, 42, 43, 44]. Furthermore, we found that urinary cells are quite similar to kidney cells, indicating renal cells were ‘flushed’ into urine during kidney injury, thus making it possible to develop non‐invasive methods for evaluating the progression and development of FSGS. Accordingly, we evaluated the key EP‐Genes distribution of FSGS patients by IHC, and validated that these genes were solely expressed by tubular cells. Quantitative analysis showed that only four genes (SPP1, SERPINA1, CLU and CST3) were drastically increased in renal tubules of FGSG patients, consistent with our bioinformatic analysis with transcriptomic data. It was reported that SPP1 [36, 45, 46], APOE [47, 48, 49, 50], SERPINA1 [51, 52, 53] and CLU [54, 55, 56] were mainly involved in innate immunity, critical for the development of FSGS [11, 57, 58, 59, 60, 61, 62] especially for the pathogenesnesis of tubular injury [63]. Innate immunity helps “arrest” circulating monocytes in the pathological sites [64]. The focal immune inflation and inflammation of glomerular may lead to remodel glomerular basement membrane [65]. At the same time, transcriptomic analysis of renal punctures from FSGS patients verified that SPP1 and ALB were consistent with the Nephroseq database; interestingly, SPP1 exhibited an AUC value > 0.8 in both datasets. To evaluate the diagnostic value of these genes in urine, we performed bulk sequencing; however, transcriptomic analysis showed only SPP1 was consistently highly expressed in urine of FSGS, and SPP1 had the highest AUC value among these key EP‐Genes. Thus, SPP1 was identified as the sole urine biomarker of FSGS.

We speculated SPP1 could serve as a valuable urine marker for the diagnosis of FSGS. Notably, we further explored the cell type of SPP1 and found that SPP1 was mainly expressed by renal tubule cells, podocytes and macrophages in FSGS. It was reported that accumulation of macrophages (most likely increased expression of SPP1) promotes renal injury and fibrotic remodeling in glomerulonephritis [66]. It has been shown that injured podocytes in FSGS [67] could be ‘flushed’ into urinary cavity, resulting in podocyte ‘depletion’, provoking the glomerular basement membrane and leading to progressive focal and segmental sclerosis [68]. These findings suggest that the immune response induced by monocyte/macrophage infiltration into the kidney is essential for FSGS.

SPP1 hallmarks profibrotic macrophage and drives renal fibrosis [36]. It was significantly correlated with macrophage infiltration and M2 polarization [69]. It has also been reported that SPP1 plays an important role in modulating macrophage immunological features [70]. In addition, SPP1 predicted prostate cancer risks in urine sediments better than previously reported biomarkers [71]. Other studies reported that urine SPP1 was a potential biomarker for Alzheimer's disease and kidney injury [72, 73]. In the present study, we further identified that, in FSGS, SPP1 was mainly derived from renal tubular cells and podocytes and detected in kidney and urine at a markedly high level. Accordingly, we proposed that urine SPP1 could serve as a biomarker for the development of FSGS. Meanwhile, urine SPP1 indicates an adverse prognostic factor. Broadly, SPP1 blockade may possess therapeutic value with respect to FSGS.

The present study had several limitations. First, all the data and biopsies used in the study were ‘cross‐sectional’. Long‐term cohort studies are required to evaluate our discoveries. In addition, the sample size in our study is limited and a larger sample size is required to validate the results. More importantly, the samples analyzed and used in the present study were mainly acquired from FSGS patients. To confirm the diagnostic values of urine SPP1, data and samples from patients suffering other renal diseases, such as membranous nephropathy, lupus, IgA nephropathy, etc., are also required. After all of the above, the present study still sheds light upon and proposes the potential diagnostic value of urine SPP1 for FSGS.

## Conflict of interest

The authors declare no conflict of interest.

### Peer review

The peer review history for this article is available at https://www.webofscience.com/api/gateway/wos/peer‐review/10.1002/2211‐5463.13704.

## Author contributions

CX, QY and WL designed the project. QY, GX, SP and BX prepared the manuscript. HL created the prognostic model. GH studied, analyzed and carried out the *in vitro* studies. XC and RY revised the manuscript. All authors contributed to data analysis and integration, visualization, and figure generation, and approved the final version of the manuscript submitted for publication.

## Supporting information


**Fig. S1.** Umap and violin plots showing expression of canonical marker genes for 12 cell clusters by integrating FSGS urine single cell with human kidney single cell datasets.
**Fig. S2.** Two scRNA‐seq datasets from the GSE131685 and GSE176465 datasets were identified genes specifically expressed, respectively.
**Fig. S3.** Expression of canonical marker genes for cell clusters of GSE131685 and GSE176465 datasets, respectively.

**Data S1.** Two gene lists encoding extracellular proteins and a gene list of scRNA‐DEGs and IMC data.

**Data S2.** Urinary raw sequencing data.

## Data Availability

The data that support the findings of this study are openly available in NCBI Gene Expression Omnibus and are accessible through https://www.ncbi.nlm.nih.gov/geo/query/acc.cgi?acc=GSE131685, GEO Series accession number [GSE131685], https://www.ncbi.nlm.nih.gov/geo/query/acc.cgi?acc=[GSE176465], GEO Series accession number [GSE176465], https://www.ncbi.nlm.nih.gov/geo/query/acc.cgi?acc=[GSE125779], GEO Series accession number [GSE125779], https://www.ncbi.nlm.nih.gov/geo/query/acc.cgi?acc=[GSE121211], GEO Series accession number [GSE121211], https://www.proteinatlas.org, https://www.uniprot.org/uploadlists/, and https://www.nephroseq.org. The raw urine cell sequencing data that support the findings of this study are available from the corresponding author upon reasonable request. Urinary raw sequencing data in this study are available in Data S2.
