# Supplementary material for: Urinary SPP1 has potential as a non‐invasive diagnostic marker for focal segmental glomerulosclerosis
Source: FEBS Open Bio. 2023 Sep 27;13(11):2061–80. doi: 10.1002/2211-5463.13704 (PMC10626280; doi:10.1002/2211-5463.13704)
Supplement: Supplementary file 1 — Fig. S1. Umap and violin plots showing expression of canonical marker genes for 12 cell clusters by integrating FSGS urine single cell with human kidney single cell datasets. Fig. S2. Two scRNA‐seq datasets from the GSE131685 and GSE176465 datasets were identified genes specifically expressed, respectively. Fig. S3. Expression of canonical marker genes for cell clusters of GSE131685 and GSE176465 datasets, respectively. [file FEB4-13-2061-s002.pdf]

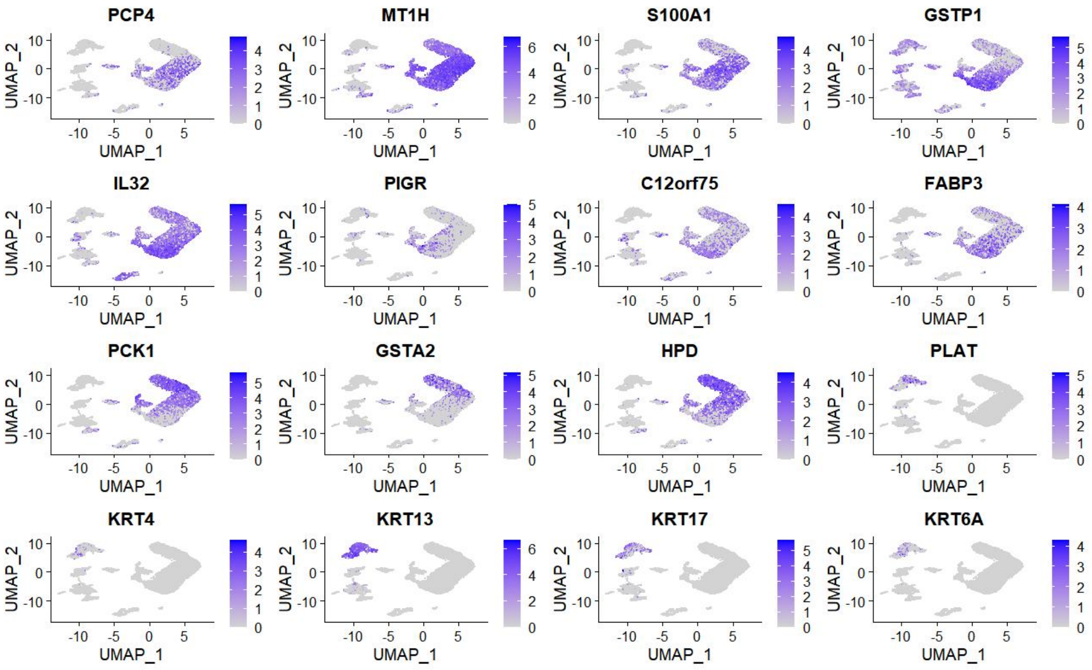
PCP4
MT1H
S100A1
GSTP1
IL32
PIGR
C12orf75
FABP3
PCK1
GSTA2
HPD
PLAT
KRT4
KRT13
KRT17
KRT6A
UMAP_1
UMAP_2

A

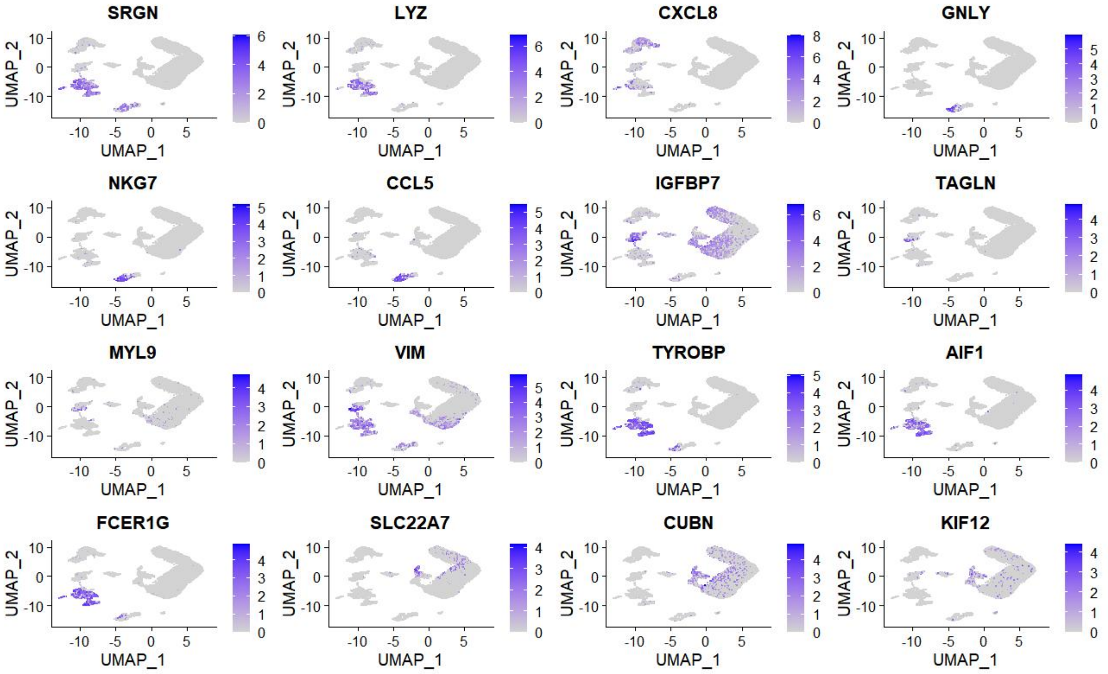
SRGN
LYZ
CXCL8
GNLY
NKG7
CCL5
IGFBP7
TAGLN
MYL9
VIM
TYROBP
AIF1
FCER1G
SLC22A7
CUBN
KIF12
UMAP_1
UMAP_2

B

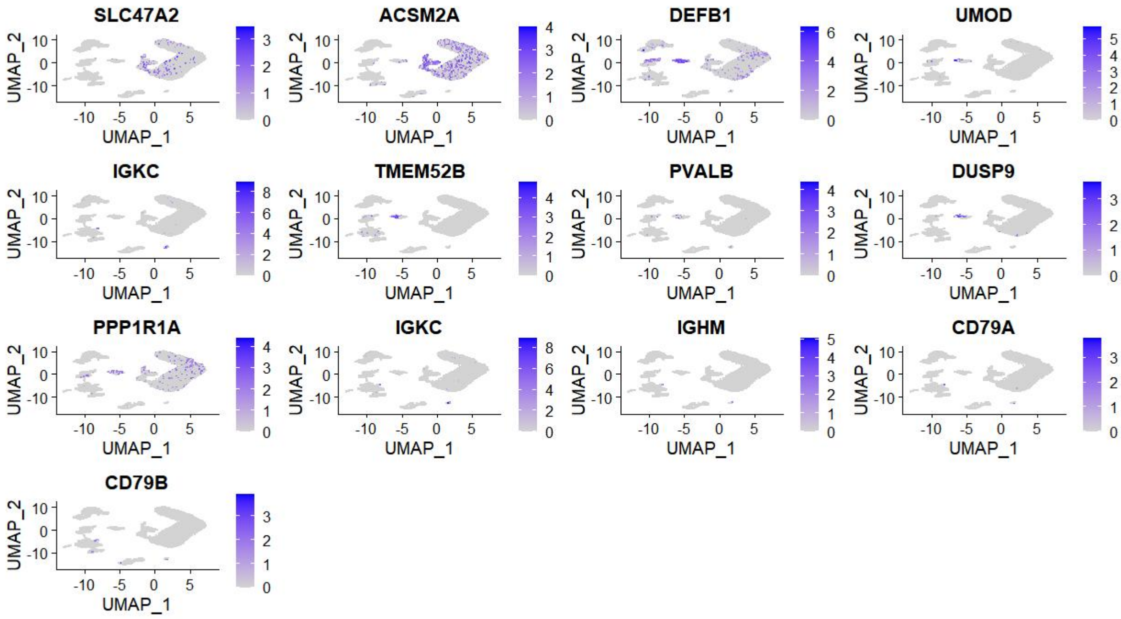
SLC47A2
ACSM2A
DEFB1
UMOD
IGKC
TMEM52B
PVALB
DUSP9
PPP1R1A
IGKC
IGHM
CD79A
CD79B
UMAP_1
UMAP_2

C

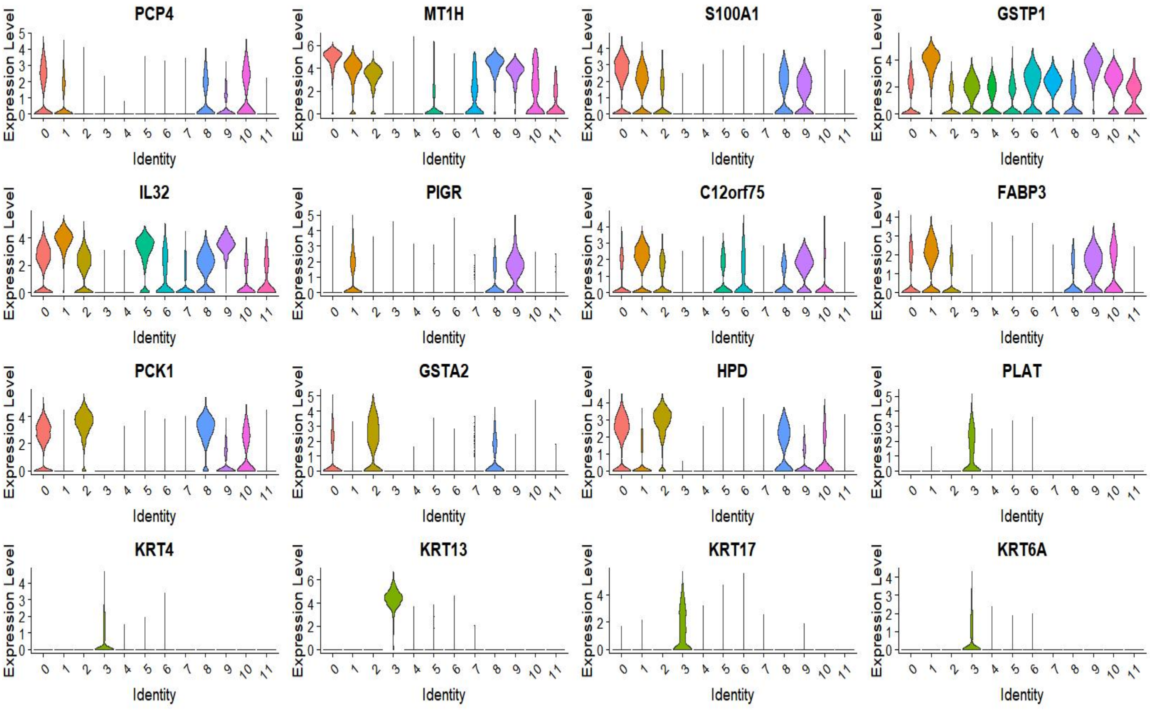
PCP4
MT1H
S100A1
GSTP1
IL32
PIGR
C12orf75
FABP3
PCK1
GSTA2
HPD
PLAT
KRT4
KRT13
KRT17
KRT6A
Expression Level
Identity

D

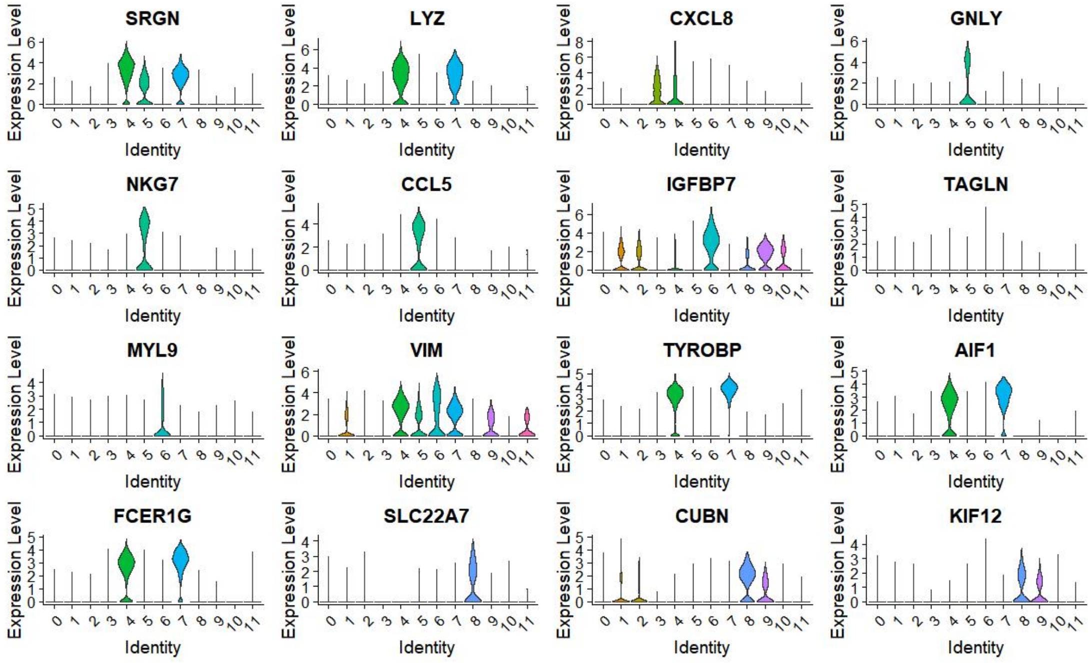

E

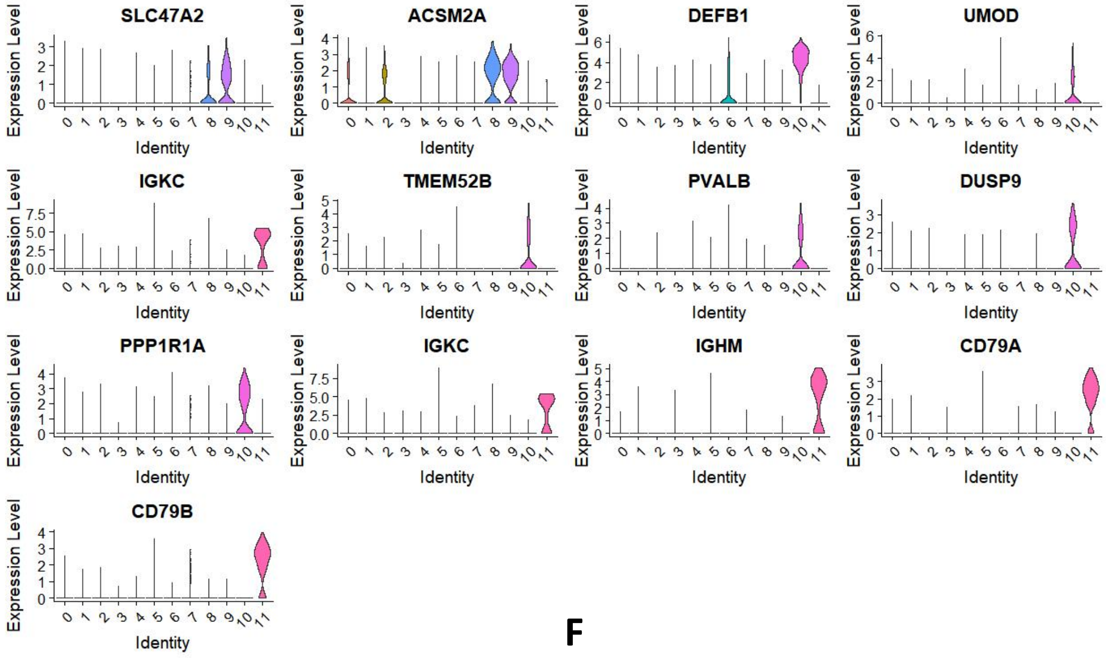

F

Supplementary Figure S1 Umap and violin plots showing expression of canonical marker genes for 12 cell clusters by Integrating FSGS urine single cell with human kidney single cell datasets. (A-C) UMAP plots showing (A) markers for proximal convoluted tubules (PCP4, MT1H and S100A), Proximal tubular_1 and Proximal tubular_2 (GSTP1, IL32, PIGR, C12orf75, FABP3,PCK1, GSTA2 and HPD) and Epithelial cells (PLAT, KRT4, KRT13, KRT17 and KRT6A) (B) markers for

Macrophages (SRGN, LYZ and CXCL8), NK cell (GNLY, NKG7 and CCL5), Fibroblasts (IGFBP7, TAGLN,MYL9, VIM), Monocyte:CD16+ (TYROPB, AIFI, PCER1G) and Proximal tubular_3 (SLC22A7, CUBN, KIF12) (C)markers for Proximal tubular_4 (SLC47A2 and ACSM2A), Distal tubular cells (DEFB1, CKB, UMOD, TMEM52B, PVALB, DUSP9, PPP1R1A, TMEM213 and PPP1R1A) and B_cell:Naive (IGKC, IGHM, CD79A and CD79B). (D-F) Violin plots showing (D) Tubular and Epithelial cell markers shown in (A-C), (E-F) immune cell markers shown in (B) and (C).

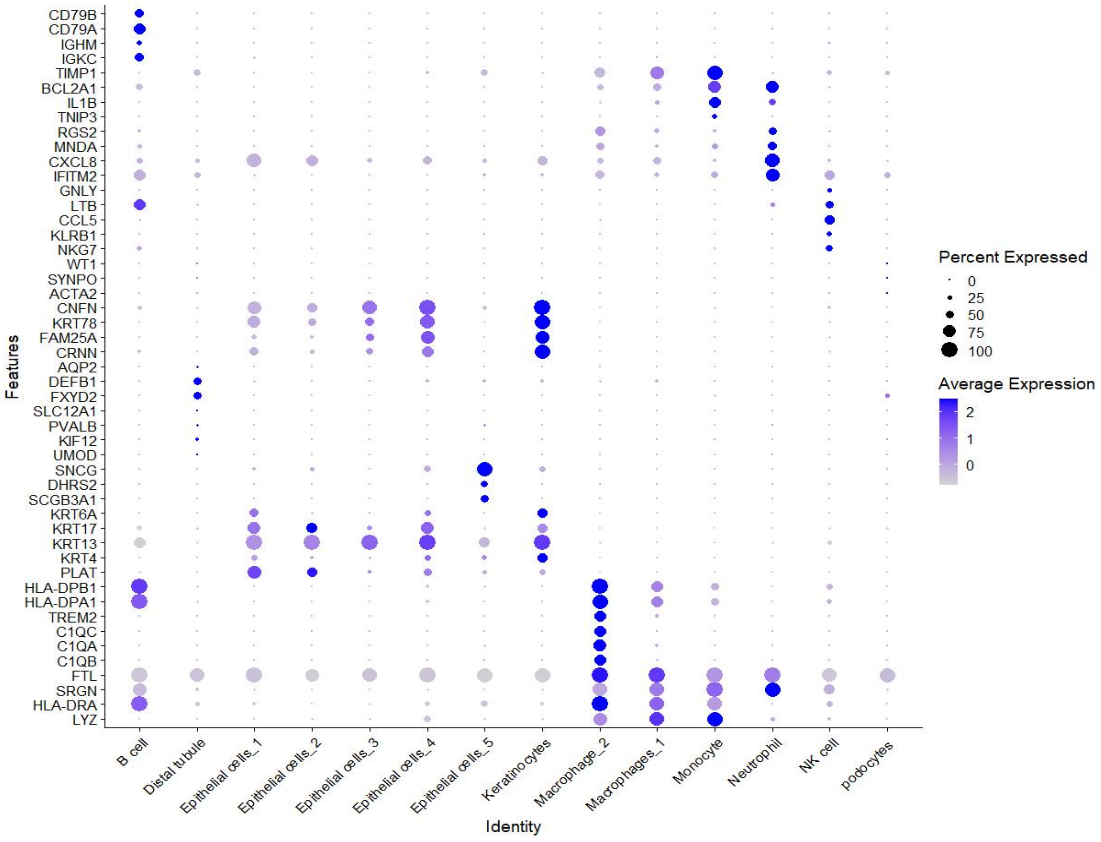
CD79B
CD79A
IGHM
IGKC
TIMP1
BCL2A1
IL1B
TNIP3
RGS2
MNDA
CXCL8
IFITM2
GNLY
LTB
CCL5
KLRB1
NKG7
WT1
SYNPO
ACTA2
CNFN
KRT78
FAM25A
CRNN
AQP2
DEFB1
FXYD2
SLC12A1
PVALB
KIF12
UMOD
SNCG
DHRS2
SCGB3A1
KRT6A
KRT17
KRT13
KRT4
PLAT
HLA-DPB1
HLA-DPA1
TREM2
C1QC
C1QA
C1QB
FTL
SRGN
HLA-DRA
LYZ
Features
B cell
Distal tubule
Epithelial cells_1
Epithelial cells_2
Epithelial cells_3
Epithelial cells_4
Epithelial cells_5
Keratinocytes
Macrophage_2
Macrophages_1
Monocyte
Neutrophil
NK cell
podocytes
Identity
Percent Expressed
0
25
50
75
100
Average Expression
2
1
0

A

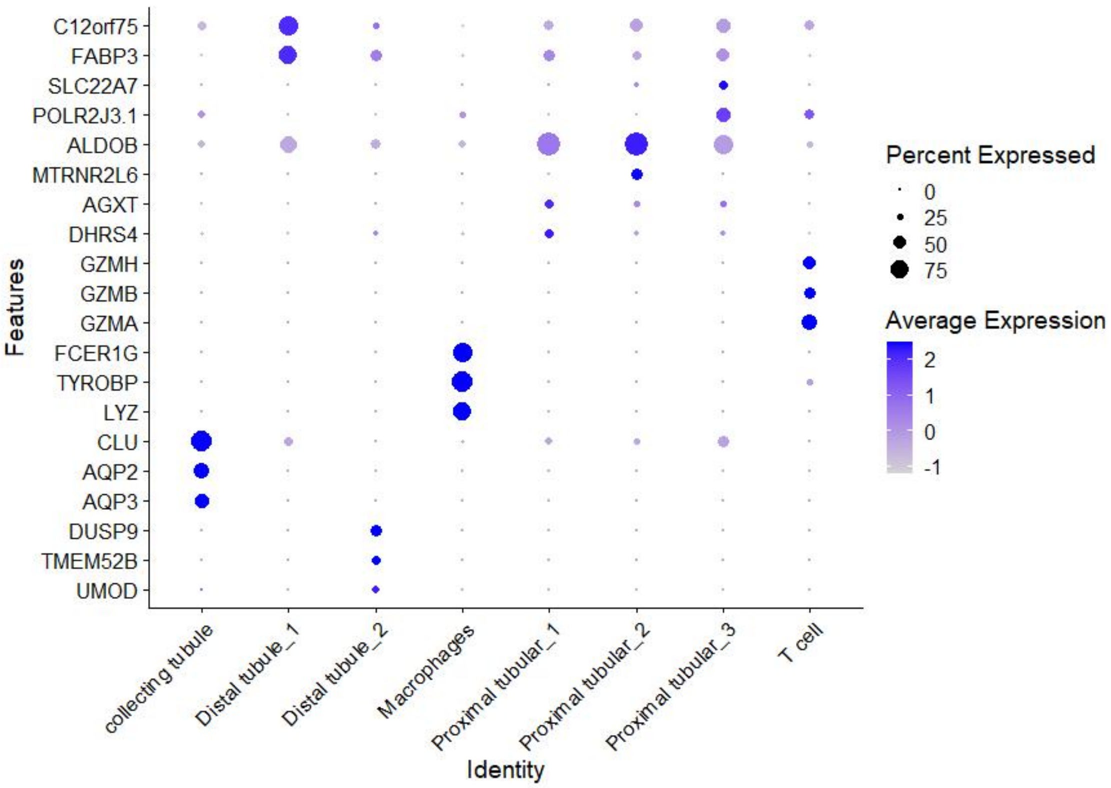
C12orf75
FABP3
SLC22A7
POLR2J3.1
ALDOB
MTRNR2L6
AGXT
DHRS4
GZMH
GZMB
GZMA
FCER1G
TYROBP
LYZ
CLU
AQP2
AQP3
DUSP9
TMEM52B
UMOD
Features
collecting tubule
Distal tubule_1
Distal tubule_2
Macrophages
Proximal tubular_1
Proximal tubular_2
Proximal tubular_3
T cell
Identity
Percent Expressed
0
25
50
75
Average Expression
2
1
0
-1

B

Supplementary Figure S2. Two scRNA-seq datasets from GSE131685 and GSE176465 datasets, were identified genes specifically expressed, respectively . (A) Bubble dot plots of the top cell-type–specific differentially expressed genes from GSE176465. (B) Bubble dot plots of the top cell-type–specific differentially expressed genes from GSE131685. The size of the dot indicates the expression percentage and the darkness of the color indicates average expression.

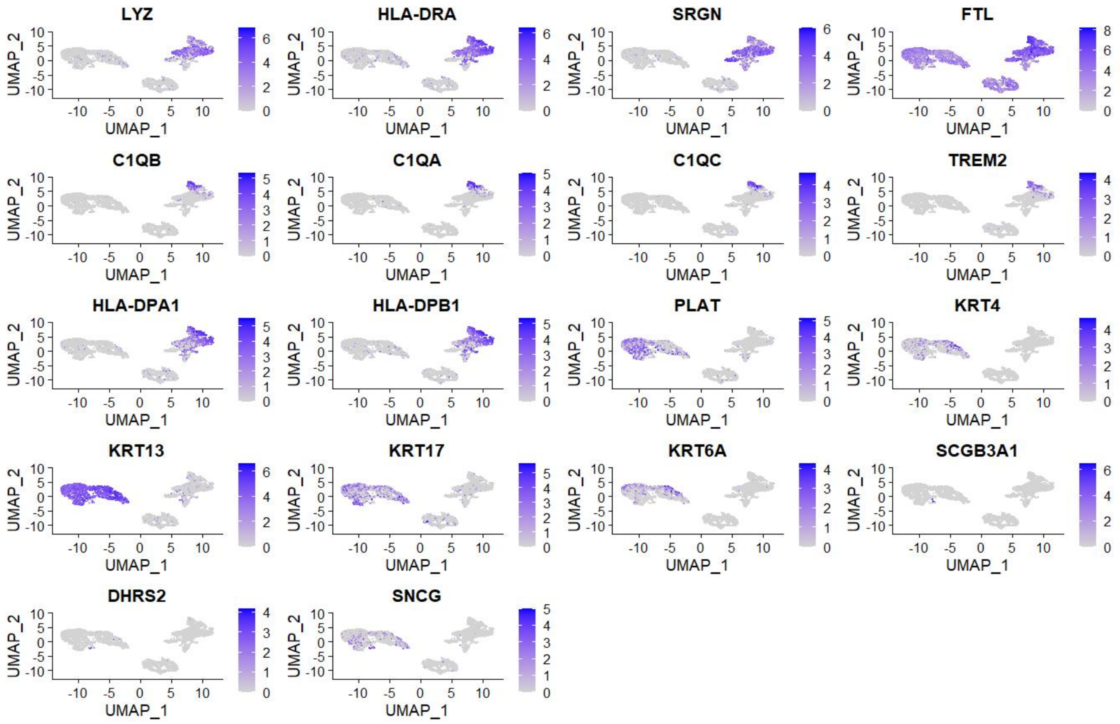
LYZ
HLA-DRA
SRGN
FTL
C1QB
C1QA
C1QC
TREM2
HLA-DPA1
HLA-DPB1
PLAT
KRT4
KRT13
KRT17
KRT6A
SCGB3A1
DHRS2
SNCG
UMAP_1
UMAP_2

A

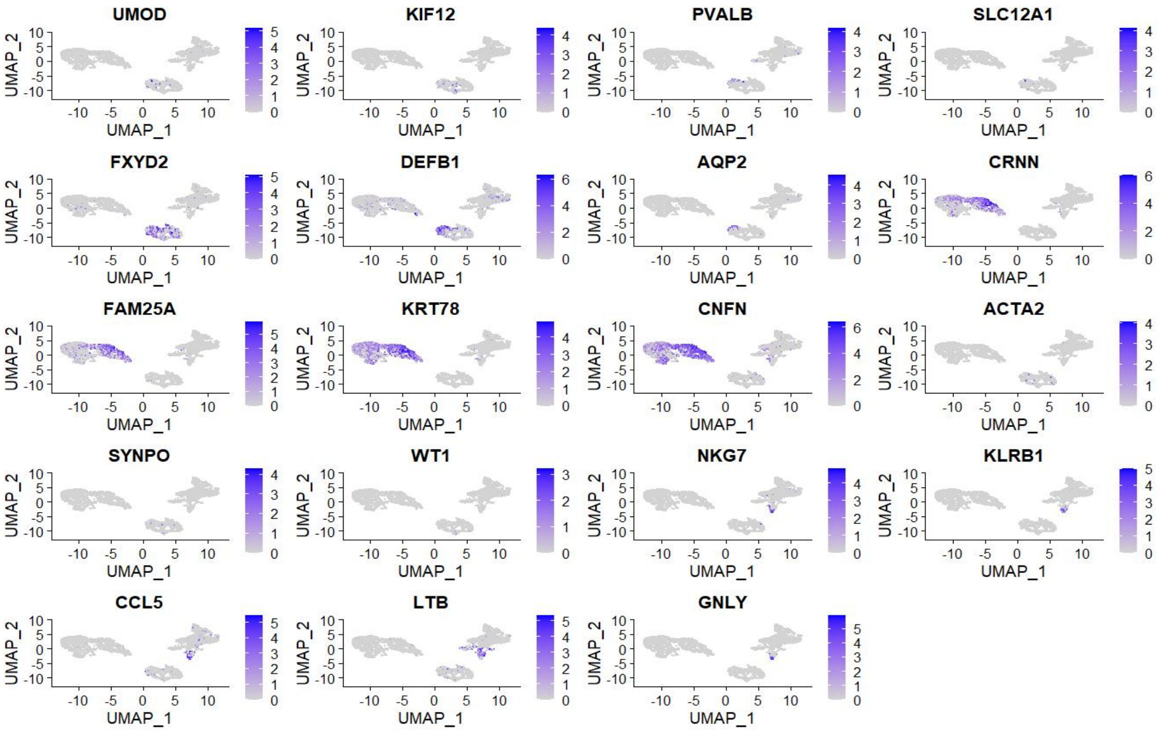
UMOD
KIF12
PVALB
SLC12A1
FXYD2
DEFB1
AQP2
CRNN
FAM25A
KRT78
CNFN
ACTA2
SYNPO
WT1
NKG7
KLRB1
CCL5
LTB
GNLY
UMAP_1
UMAP_2

B

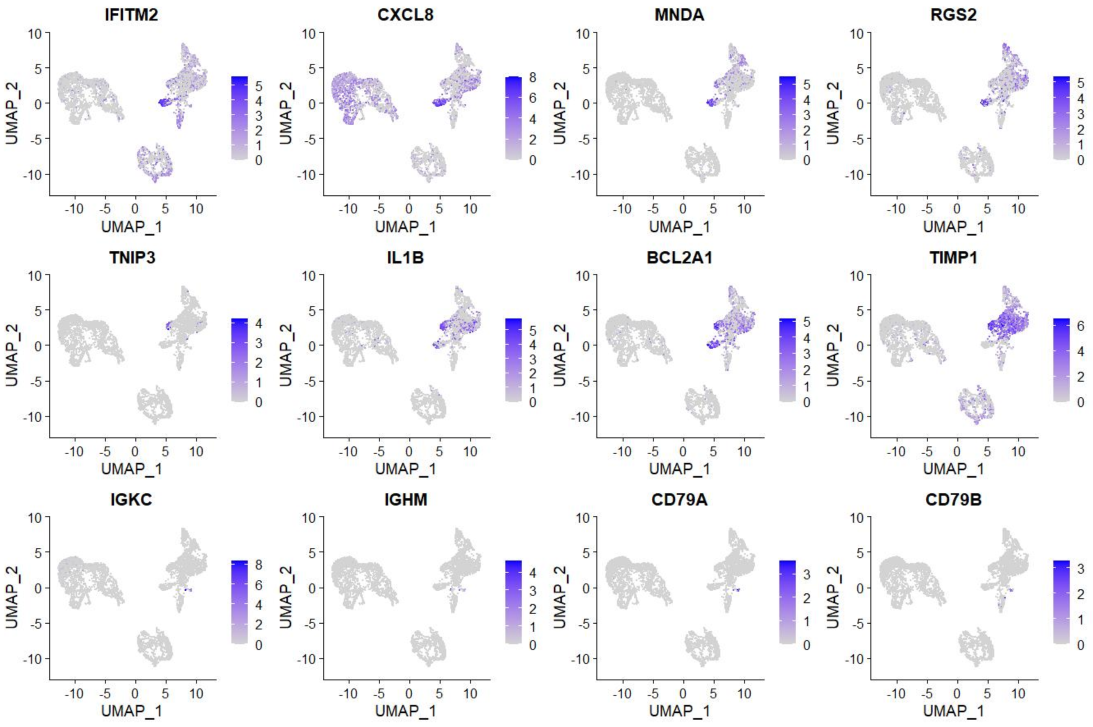
IFITM2
CXCL8
MNDA
RGS2
TNIP3
IL1B
BCL2A1
TIMP1
IGKC
IGHM
CD79A
CD79B
UMAP_1
UMAP_2

C

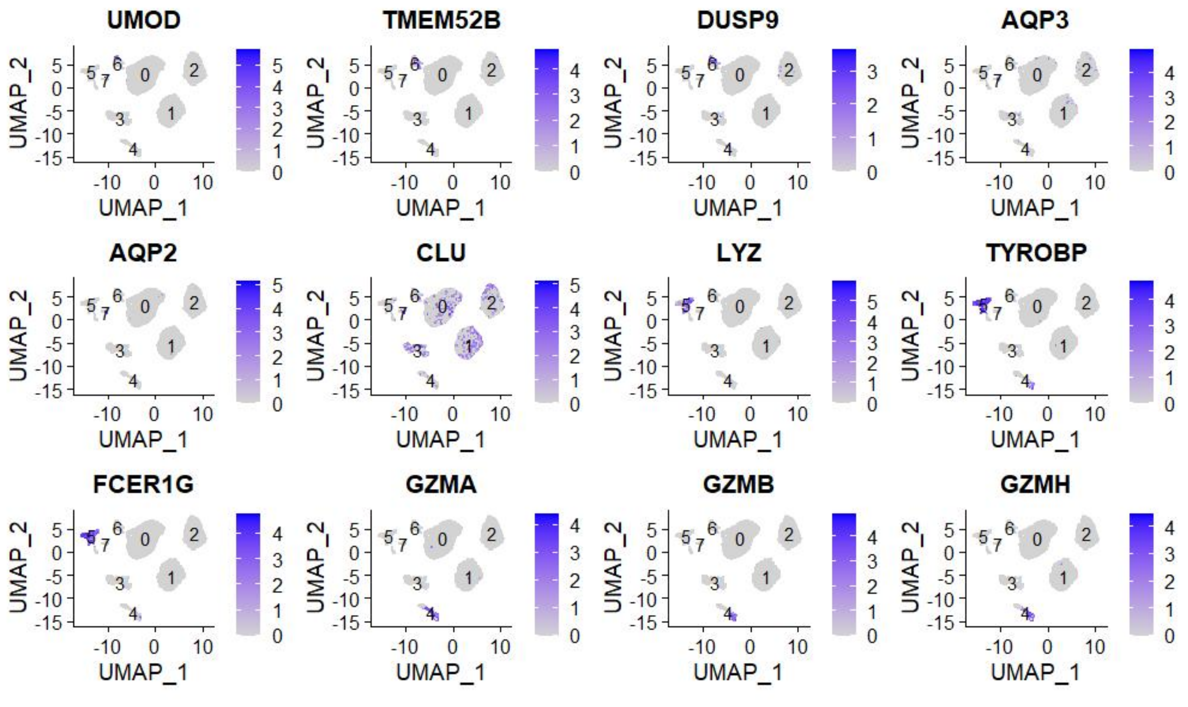
UMOD
TMEM52B
DUSP9
AQP3
AQP2
CLU
LYZ
TYROBP
FCER1G
GZMA
GZMB
GZMH
UMAP_1
UMAP_2

D

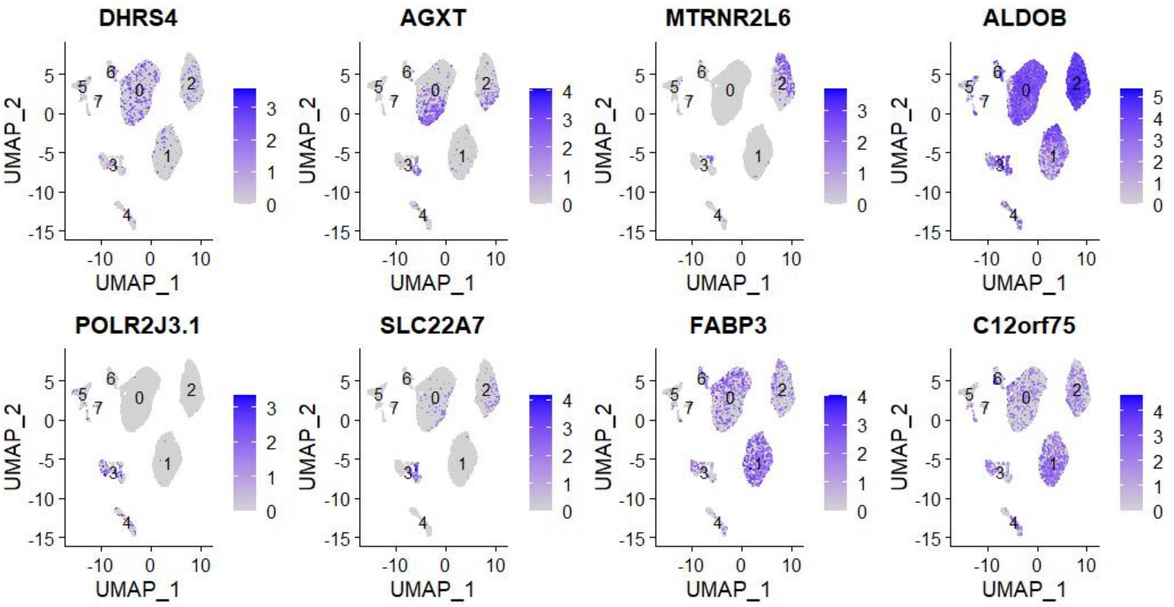

E

Supplementary Figure S3. Expression of canonical marker genes for cell clusters of GSE131685 and GSE176465 datasets, respectively. (A-C) UMAP plots from GSE176465 showing (A) markers for Macrophages (LYZ, HLA-DRA, SRGN and FTL) and urogenital epithelial cells (C1QB, C1QA, C1QC, TREM2, HLA-DPA1, HLA-DPB1, PLAT, KRT4, KRT13, KRT17, KRT6A, SCGB3A1, DHRS2 and SNCG) (B) markers for Distal tubular cells (UMOD, KIF12, PVALB, SLC12A1, FXYD2, DEFB1and AQP2), Keratinocytes (CRNN, FAM25A, KRT78 and CNFN), Podocytes (ACTA2, SYNPO, WT1) and NK cells (NKG7, KLRB1, CCL5, LTB and GNLY). (C) markers for Neutrophil (IFITM2, CXCL8, MNDA and RGS2), Monocyte (TNIP3, IL1B, BCL2A1 and TIMP1) and B cells (GKC, IGHM, CD79A and CD79B). (D-E) UMAP plots from GSE131685 showing the 8 cell clusters where highly variable genes are located.
